# Proton‐Driven Transformable ^1^O_2_‐Nanotrap for Dark and Hypoxia Tolerant Photodynamic Therapy

**DOI:** 10.1002/advs.202200128

**Published:** 2022-04-18

**Authors:** Dapeng Chen, Hanming Dai, Weili Wang, Yu Cai, Xiaozhou Mou, Jianhua Zou, Jinjun Shao, Zhengwei Mao, Liping Zhong, Xiaochen Dong, Yongxiang Zhao

**Affiliations:** ^1^ Clinical Research Institute Zhejiang Provincial People's Hospital Affiliated People's Hospital Hangzhou Medical College Hangzhou 310014 P. R. China; ^2^ Key Laboratory of Flexible Electronics (KLOFE) & Institute of Advanced Materials (IAM) Nanjing Tech University (NanjingTech) Nanjing 211816 P. R. China; ^3^ MOE Key Laboratory of Macromolecular Synthesis and Functionalization Department of Polymer Science and Engineering Zhejiang University Hangzhou 310027 P. R. China; ^4^ National Center for International Biotargeting Theranostics Guangxi Key Laboratory of Biotargeting Theranostics Collaborative Innovation Center for Targeting Tumor Theranostics Guangxi Medical University Guangxi 530021 P. R. China

**Keywords:** ^1^O_2_‐nanotrap, hypoxic tumor, photodynamic therapy, photosensitizer

## Abstract

Despite the clinical potential, photodynamic therapy (PDT) relying on singlet oxygen (^1^O_2_) generation is severely limited by tumor hypoxia and endosomal entrapment. Herein, a proton‐driven transformable ^1^O_2_‐nanotrap (ANBDP NPs) with endosomal escape capability is presented to improve hypoxic tumor PDT. In the acidic endosomal environment, the protonated ^1^O_2_‐nanotrap ruptures endosomal membranes via a “proton‐sponge” like effect and undergoes a drastic morphology‐and‐size change from nanocubes (≈94.1 nm in length) to nanospheres (≈12.3 nm in diameter). Simultaneously, anthracenyl boron dipyrromethene‐derived photosensitizer (ANBDP) in nanospheres transforms to its protonated form (ANBDPH) and switches off its charge‐transfer state to achieve amplified ^1^O_2_ photogeneration capability. Upon 730 nm photoirradiation, ANBDPH prominently produces ^1^O_2_ and traps generated‐^1^O_2_ in the anthracene group to form endoperoxide (ANOBDPH). Benefitting from the hypoxia‐tolerant ^1^O_2_‐release property of ANOBDPH in the dark, the ^1^O_2_‐nanotrap brings about sustained therapeutic effect without further continuous irradiation, thereby achieving remarkable antitumor performance.

## Graphical abstract

A ^1^O_2_‐nanotrap is prepared to combat hypoxic tumors. In proton‐rich endosomal environment, the ^1^O_2_‐nanotrap can escape from endosomal entrapment by “proton‐sponge”‐like effect. Simultaneously, the ^1^O_2_‐nanotrap undergo morphology‐and‐size transformation from nano‐cubes to nano‐spheres, allowing amplified PDT efficacy. Harnessing the reversible ^1^O_2_ capture‐and‐release property, the ^1^O_2_‐nanotrap bring sustained therapeutic effect without further continuous irradiation, which significantly enhance the antitumor efficacy.

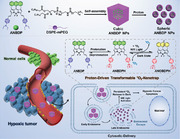

## 1 Introduction

Photodynamic therapy (PDT), a therapeutic modality relying on photo‐triggered reactive oxygen species (ROS), has emerged as a promising approach for cancer treatment.^[^ ^1^ ^]^ Essentially, a PDT process involves the administration of phototherapeutic agents followed by local non‐ionizing irradiation for producing cytotoxic ROS (e.g.,^1^O_2_, HO^●^, O_2_^●–^).^[^ ^2^ ^]^ Among phototherapeutic agents, the application of nanoagents has been an extremely promising avenue in resolving some shortcomings associated with classic molecular photosensitizers in terms of their targeting and delivery characteristics.^[^ ^3^ ^]^ In addition to receptor‐mediated endocytosis, most nanoparticles (NPs) can be taken into cells through endosomal trafficking.^[^ ^4^ ^]^ However, a common issue comes that the entrapment of NPs within endosomes is undesirable for PDT applications.^[^ ^5^ ^]^ Since the maturation of early endosomes into late endosomes is characterized by rapid acidification and recruitment of degradative enzymes to digest the entrapped contents, which may severely discount the ROS generation efficiency of nanoagents.^[^ ^6^ ^]^ To achieve better PDT efficacy, it is anticipated to engineer the nanoagents with ability to escape from endosome entrapment.^[^ ^7^ ^]^ Toward this end, some nanoagents coated with viral capsids, endosomal fusible membranes, or polyethyleneimine (PEI) have been reported with endosomal escape ability.^[^ ^6^ ^]^ However, the complexity and cost of preparing viral vectors or membrane envelope limit their further biomedical application.^[^ ^6^ ^]^ The cytotoxicity and poor stability in biological medium are the two major concerns for the use of PEI. Therefore, it is desired to economically develop biocompatible and biological stable nanoagents that can escape from endosomal entrapment for PDT treatment.

In addition to endosomal escape capability, morphology and size both determine the performance of nanoagents in complicated biological systems.^[^ ^8^ ^]^ For example, worm‐like nanomicelles may achieve long blood circulation but poor cellular uptake.^[^ ^9^, ^10^ ^]^ NPs with a size less than 20 nm are easier to penetrate tumors and cross in vivo or intracellular barriers, such as blood–brain barrier (BBB) and nuclear membrane of cells.^[^ ^9^, ^11^, ^12^ ^]^ Nevertheless, they also exhibit a relatively short tissue retention period, which is not favorable for in vivo imaging or treatment.^[^ ^8^ ^]^ Thus, it is challenging to select a morphology or size that can meet all the delivery requirements. In this regard, morphology‐and‐size transformable PDT nanoagents with endosomal escape capability show great promise, as they can deform nanoagents for triggering ROS generation with spatiotemporal control, bringing out improved PDT efficacy, increased tumor accumulation, and reduced side‐effects.^[^ ^12^ ^]^

To a large extent, PDT undergoes energy‐transfer based type II pathway to generate cytotoxic singlet oxygen (^1^O_2_). The generated ^1^O_2_ will cause irreversible damage to cancer cells and microvasculatures, as well as induce an inflammatory and immune response.^[^ ^13^ ^]^ However,^1^O_2_‐based PDT is highly dependent on O_2_ concentration, and this greatly discounts its therapeutic efficacy against tumor cells in interior hypoxic regions (PaO_2_ < 5 mm Hg).^[^ ^14^ ^]^ What's even worse, continuous irradiation during PDT process will expedite O_2_‐depletion and exacerbate the hypoxia level in exterior tumor regions.^[^ ^15^ ^]^ Therefore, hypoxia is usually considered as the “Achilles’ heels” of ^1^O_2_‐mediated PDT.^[^ ^16^ ^]^ Among various approaches tackling the O_2_‐shortage issue of type II PDT, O_2_‐replenishing strategy has been demonstrated to be available and effective for alleviating tumor hypoxia. This method usually employs O_2_‐nanocarriers (e.g., fluorocarbon nanovesicles^[^ ^17^ ^]^, hemoglobin nanoformulations^[^ ^18^ ^]^) or O_2_‐evolving materials (e.g., MnO_2_,^[^ ^19^ ^]^ MnFeO_4_,^[^ ^20^ ^]^ and catalase^[^ ^21^ ^]^) to directly or indirectly increase the O_2_ concentration in solid tumors. Nevertheless, the therapeutic efficacy of fluorocarbon‐based materials may be impaired by poor O_2_‐loading efficiency and off‐target O_2_ leakage in blood circulation. Moreover, the administration of catalytic materials may suffer from inadequate H_2_O_2_ concentration (<50* × *10^‐6^ m) in tumor microenvironment.^[^ ^22^ ^]^ Hence, it is still a challenging conundrum to develop new methods on reversing the low therapeutic efficacy of PDT against hypoxic tumors.

**Scheme 1:**
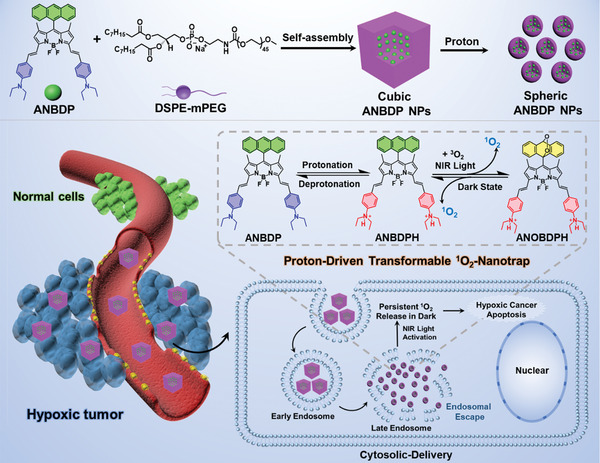
The preparation of proton‐driven transformable ^1^O_2_‐nanotrap and its cytosolic delivery for hypoxic cancer PDT.

Considering that ^1^O_2_ is the ultimate cytotoxic product of the PDT process, a ^1^O_2_‐trap that can serve as the host to capture ^1^O_2_ under irradiation and chemically release ^1^O_2_ during the subsequent dark period will provide a sustainable cell‐killing effect neglecting the PDT‐induced temporary hypoxia. As shown in **Scheme** **1**, we have devised a proton‐driven transformable ^1^O_2_‐nanotrap ANBDP NPs as a novel dark‐and‐hypoxia tolerant PDT agent, which was achieved by encapsulating a well‐designed photosensitizer in 1,2‐distearoyl‐sn‐glycero‐3‐phosphoethanolamine‐polyethyleneglycol (DSPE‐mPEG_2000_). Boron dipyrromethene (BODIPY) are employed as the biocompatible photosensitizer because they inherent excellent optical properties, such as sharp absorption and emission spectra, high fluorescence quantum yields, high thermal‐stability, and photochemical stability. More importantly, it is easy to tune the structure of BODIPY to afford desired near‐infrared absorption/fluorescence as well as ^1^O_2_ generation. The anthracenyl BODIPY‐derived photosensitizer (ANBDP) encapsulated in nanoparticles (NPs) is molecularly conjugated with proton‐sensitive moieties (diethylamino phenyl groups) and a ^1^O_2_‐trap utilizing anthracenyl group, which has been reported to react with ^1^O_2_ in its middle aromatic ring to form the corresponding endoperoxides.^[^ ^23^ ^]^ In the proton‐rich environment, the ^1^O_2_‐nanotrap physically disrupts the endosomal membrane through a “proton‐sponge”‐like effect. It simultaneously undergoes a dramatic morphology‐and‐size transformation from cube‐like NPs (≈94.1 nm in length) into sphere‐like NPs (≈12.3 nm in diameter), accompanied by a 15.8‐fold amplification of ^1^O_2_ photogeneration. Triggered by a 730 nm laser, the protonated photosensitizer (ANBDPH) in ANBDP NPs can produce ^1^O_2_ prominently. The as‐generated ^1^O_2_ in its anthracenyl group can react with 1,3‐dienes in the middle carbonyl ring of anthracenyl group via Dies–Alder addition reaction, “trapping” ^1^O_2_ to form endoperoxide (ANOBDPH). Without further continuous irradiation, the endoperoxide product is thermodynamically, structurally unstable and undergoes cycloreversion retro‐Diels‐Alder reaction in the physiological environment, enabling reversible “release” of ^1^O_2_ without any side reactions in a dark‐tolerant manner.^[^ ^24^ ^]^ In vivo study shows that the prolonged ^1^O_2_‐release of ANBDP NPs significantly enhances its therapeutic efficacy against hypoxic tumors (96.7% regression rate). This design of transformable ^1^O_2_‐nanotrap sheds new light on hypoxic cancer PDT.

## 2 Results and Discussion

**Figure 1:**
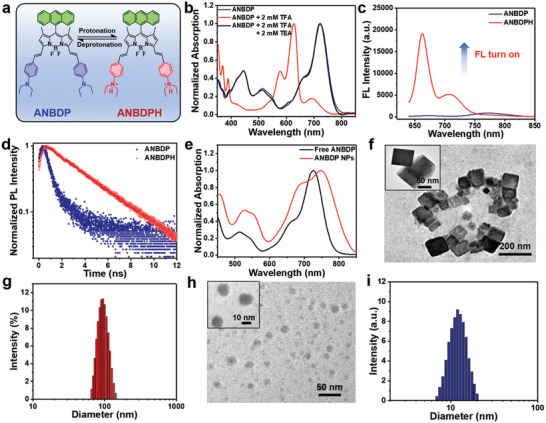
a) Protonation and deprotonation process of ANBDP. b) Normalized absorption spectra of ANBDP upon the addition of TFA and TEA. c) Fluorescence (FL) spectra of ANBDP and ANBDPH at 5 × 10^‐6^ m. d) Transient fluorescence lifetime of ANBDP and ANBDPH. e) Normalized absorption of free ANBDP and ANBDP NPs. f,h) TEM images of ANBDP NPs at pH 7.4 and 5.0. g,i) DLS size distribution of ANBDP NPs at pH 7.4 and 5.0.

The general synthetic routes for ANBDP and its contrast photosensitizers (BDP and NBDP) are shown in Figure S1 (Supporting Information). To investigate the reversible protonation process of ANBDP (**Figure** **1**a), trifluoroacetic acid (TFA) and triethylamine (TEA) were employed to protonate and deprotonate ANBDP, respectively. As shown in Figure 1b, free ANBDP in tetrahydrofuran (THF) presents an absorption band ranging from 600 to 800 nm, with an absorption peak at 723 nm. Upon the addition of TFA, the diethylamino phenyl groups in ANBDP received two protons to give ANBDPH, with blue‐shifted absorption peak at 627 nm. When TEA was added to deprotonate ANBDPH, the absorption peak underwent a bathochromic shift from 627 to 723 nm, demonstrating the reversible protonation process of ANBDP photosensitizer. The fluorescence spectra in Figure 1c showed a stronger fluorescence emission of ANBDPH than ANBDP, which denoted that the radiative fluorescence pathway of the excited ANBDPH was switched on. Moreover, fluorescence lifetime of ANBDPH was measured to be 2.32 ns (Figure 1d), much longer than that of ANBDP (0.54 ns). Thus, we propose a switchable charge‐transfer mechanism for the drastic fluorescence change phenomenon.^[^ ^25^ ^]^ The highest occupied molecular orbital (HOMO) and lowest unoccupied molecular orbital (LUMO) from ANBDP lie in diethylamino phenyl units and dipyrrometheneboron difluoride moiety, respectively. The HOMO of the dipyrrometheneboron difluoride moiety is higher than that of diethylamino phenyl groups. When electrons are photo‐excited, they tend to fall into the HOMO of diethylamino phenyl group rather than the boron–pyrrole moiety, thus forming a charge‐transfer (CT) state and inhibiting fluorescence emission (Figure S2a, Supporting Information). Once ANBDP is protonated to ANBDPH, HOMO of the boron–pyrrole moiety is lower than that of diethylamino phenyl groups, and the photo‐excited electrons tend to fall into HOMO of dipyrrometheneboron difluoride moiety rather than diethylamino phenyl group, whereby CT state turns off, and the fluorescence emission channel turns on (Figure S2b, Supporting Information). Meanwhile, due to the enhanced intersystem crossing (ISC) of ANBDPH, more triplet state (T_1_) can be generated after protonation, thus enabling itself to efficiently sensitize ^3^O_2_ for ^1^O_2_ production.

Through self‐assembly technique, photosensitizer ANBDP was encapsulated in amphiphilic polymer DSPE‐mPEG_2000_ to form ANBDP NPs. Notably, as depicted in Figure 1e, the as‐prepared ANBDP NPs presented a longer wavelength (748 nm) of absorption peak than that of free ANBDP (723 nm), indicating the existence of *π*–*π* interactions among ANBDP molecules in ANBDP NPs. Meanwhile, both BDP NPs and ANBDP NPs exhibited absorption and fluorescence emission above 600 nm, denoting their potency for fluorescence imaging‐guided cancer therapy (Figures S3 and S4, Supporting Information). As demonstrated by the transmission electron microscopy (TEM) images in Figure 1f, ANBDP NPs in water at pH 7.4 showed a cubic morphology. Dynamic light scattering (DLS) results showed that the size distribution of cubic ANBDP NPs is 94.1 ± 12.3 nm. However, when the medium changes into an acetic environment, the cubic‐like ANBDP NPs presented a drastic transformation into nanospheres (Figure 1h) with a size distribution of 12.3 ± 6.7 nm (Figure 1g). We changed the pH value of medium from 7.4 to 5.0 and further investigated the morphology of ANBDP NPs at different point via TEM. The results (Figure S5a, Supporting Information) indicated that ANBDP NPs undergoes dynamic changes from nanocubes to nanospheres in 6 h. The transformation could be attributed to the hydrophilicity change of ANBDP photosensitizer during the protonation process. The hydrophilicity of positive charged ANBDPH was better than neutral charged ANBDP, which significantly affected intermolecular interactions among encapsulated photosensitizers and contributed to the morphology‐and‐size change of ANBDP NPs. Moreover, after 4 weeks of storage, no abnormal size changes were observed, confirming the excellent long‐term stability of nanoagents (Figure S5, Supporting Information).

**Figure 2:**
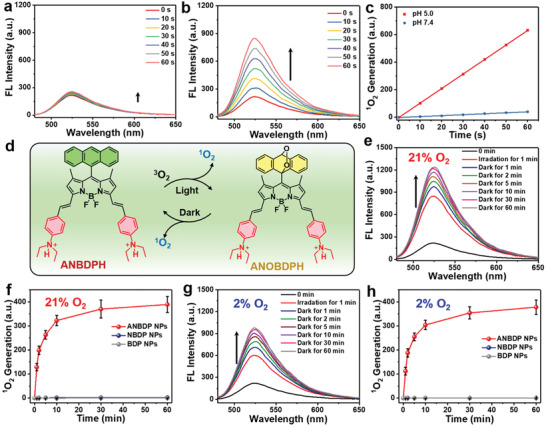
a) Fluorescence intensity change of SOSG triggered by ANBDP NPs at pH 7.4 under irradiation. b) Fluorescence intensity change of SOSG triggered by ANBDP NPs at pH 5.0 under irradiation. c) ^1^O_2_ generation capability of ANBDP NPs at pH 5.0 and 7.4 as indicated by SOSG. d) Schematic illustration of the reversible ^1^O_2_ capture and release of ANBDPH under irradiation and dark condition. e,f) Fluorescence intensity change of SOSG in ANBDP NPs (pH 5.0) triggered by laser irradiation for 1 min and dark for 60 min under normoxia (21% O_2_) environment. g,h) Fluorescence intensity change of SOSG in ANBDP NPs (pH 5.0) triggered by laser irradiation for 1 min and dark for 60 min under hypoxia (2% O_2_) environment. (Laser: 730 nm, 0.05 W cm^–2^).

It was envisioned that the proton‐driven transformation would enhance the ^1^O_2_ generation efficiency of ANBDP NPs. Therefore, the ^1^O_2_ generation of ANBDP NPs in different conditions was qualitatively or quantitatively investigated by utilizing singlet oxygen sensor green (SOSG) as the ^1^O_2_‐specific probes. Without light irradiation, ANBDP NPs could not produce any ^1^O_2_ (Figure S7a, Supporting Information). Triggered by 730 nm light, ANBDP NPs presented weak ^1^O_2_ photogeneration capability at pH 7.4 (**Figure** **2**a). While in the acidic conditions (pH 5.0), ANBDP NPs triggered a 15.8‐fold stronger fluorescence intensity increase to SOSG, which denoted the proton‐specific ^1^O_2_ photogeneration capability of ANBDP NPs. The kind of photogenerated ROS was further confirmed as ^1^O_2_ by electron spin resonance (ESR) test (Figure S6a, Supporting Information), whereas no signals of HO^●^ or O_2_^●–^ could be found (Figure S6b, Supporting Information). Due to the decoration of the diethylamino phenyl group, the control agent NBDP NPs also presented an acidic triggered ^1^O_2_ photogeneration capability (Figure S7b,c in Supporting Information), while that of BDP NPs was negligible (Figure S7d,e in Supporting Information).

Because it was proposed that anthracenyl groups in ANBDP NPs could serve as the host to chemically capture and release ^1^O_2_ in a hypoxia‐tolerant manner (Figure 2d), we then investigated the ^1^O_2_ generation properties of NBDP NPs and ANBDP NPs in the dark conditions under both normoxia and hypoxia environment. For NBDP NPs group (Figure S8a,b in Supporting Information), fluorescence intensity of SOSG increased after 60 s irradiation, while no fluorescence change could be found in the dark conditions under both normoxia and hypoxia. By contrast, for ANBDP NPs under normoxia, the fluorescence intensity of SOSG after 60 s irradiation continued to increase over time even without photoirradiation (Figure 2e,f), which suggested that the anthracenyl group played a key role for ^1^O_2_‐release in the dark state. Notably, for ANBDP NPs under hypoxia (Figure 2g,h), the fluorescence intensity increase in the dark state of SOSG was comparable to that of normoxia groups, indicating that ANBDP NPs could effectively release ^1^O_2_ in the hypoxia state. Therefore, ANBDP NPs showed a promise for cancer PDT in a dark‐and‐hypoxia tolerant manner.

**Figure 3:**
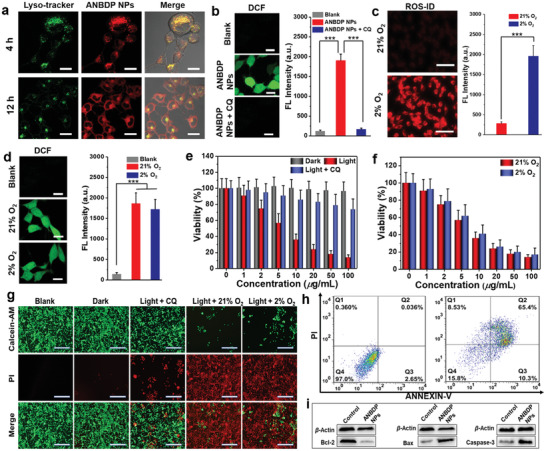
a) Confocal fluorescence imaging on endosomal entrapment of ANBDP NPs at 4 h and 12 h post‐incubation as indicated by LysoTracker green, scale bars: 10 µm. b) Confocal fluorescence imaging on intracellular ^1^O_2_‐photogeneration under different treatments as indicated by DCFH‐DA probe. Scale bars: 10 µm (*n* = 5, mean ± SD, ***p < 0.001, *t*‐test). c) Intracellular oxygen concentration as indicated by hypoxia fluorescence probe ROS‐ID. Scale bars: 50 µm (*n* = 5, mean ± SD, ***p < 0.001, *t*‐test). d) Confocal fluorescence imaging on intracellular ^1^O_2_‐generation in the dark state under 21% O_2_ and 2% O_2_ as indicated by DCFH‐DA probe. Scale bars: 10 µm (*n* = 5, mean ± SD, ***p < 0.001, *t*‐test). e) Viabilities of 4T1 cells incubated with ANBDP NPs under dark, light, light + CQ conditions (*n* = 5, mean ± SD). f) Viabilities of 4T1 cells incubated with ANBDP NPs under normoxia and hypoxia with irradiation (*n* = 5, mean ± SD). g) Fluorescence imaging of living and dead cells stained by Calcein‐AM and PI, respectively. Scale bars: 50 µm. h) Flow cytometry assay on 4T1 cells incubated with 100 µg mL^–1^ of ANBDP NPs (2% O_2_) without (left) and with irradiation (right). i) Western‐blotting assay of apoptosis‐relevant proteins in hypoxic 4T1 cells with or without ANBDP NPs treatment.

Since endosomal entrapment would severely limit the therapeutic efficacy of nanoagents, we performed subcellular distribution experiments to further investigate the endosomal escape capability of ANBDP NPs. At 4 h post‐administration of ANBDP NPs, the fluorescence of LysoTracker overlapped well with that of ANBDP NPs (**Figure** **3**a), indicating that ANBDP NPs were wrapped in the endosomes at this stage. However, at 12 h post‐administration of ANBDP NPs, the fluorescence of LysoTracker did not overlap well with that of ANBDP NPs, whose fluorescence was distributed in the whole cytoplasm of 4T1 cells. This could be attributed to the “proton‐sponge”‐like effect of the diethylamino phenyl groups in ANBDP NPs: the ability to absorb protons and induce osmotic pressure in the acidic endosomal microenvironment. The osmotic pressure destabilized and eventually disrupted endosomal membranes, thus releasing transformed ANBDP NPs into the cytoplasm. Motivated by this, we then investigated the in vitro ^1^O_2_‐photogeneration capability of ANBDP NPs. As shown in Figure 3b, no fluorescence of DCF could be found for the cells of the blank group. For groups of ANBDP NPs with irradiation, the bright fluorescence signal of DCF was found in 4T1 cells, which meant that remarkable ^1^O_2_ was generated by ANBDP NPs under irradiation. However, for ANBDP NPs group pretreated with chloroquine (CQ, increasing the pH in endosomes), negligible fluorescence of DCF was found in the cytoplasm, suggesting the enhancement of endosomal pH impeded the cytosolic ^1^O_2_‐photogeneration capacity of ANBDP NPs. Due to proton‐sensitive moieties, 4T1 cells incubated with NBDP NPs were also witnessed with remarkable ^1^O_2_ photogeneration (Figure S9, Supporting Information), while that of BDP NPs was negligible because they are lack proton‐sensitive groups. To further determine the physicochemical properties of ANBDP NPs, we investigated the ^1^O_2_ photogeneration of ANBDP NPs under 21% O_2_ and 2% O_2_. It showed that ANBDP NPs would produce more ^1^O_2_ in normoxia condition than that in hypoxia condition (Figure S10, Supporting Information), which was also in consent with results in Figure 2e,g. Considering that ANBDP NPs could chemically release ^1^O_2_ in a hypoxia‐tolerant manner, we then investigated the intracellular ^1^O_2_‐generation in the dark state under both normoxia and hypoxia. ROS‐ID, which can emit red fluorescence in hypoxic conditions (Figure 3c), was employed as the hypoxia‐specific probe for in vitro experiments. As shown in Figure 3d, without further continuous light‐irradiation, remarkable ^1^O_2_ generation could be found under both 21% O_2_ and 2% O_2_ environments, manifesting that ANBDP NPs could be utilized as robust ^1^O_2_‐nanotrap for hypoxia‐tolerant PDT in the dark state. To ensure the effects of anthracene group, BDP‐2 NPs containing only anthracene group were also monitored under dark state (Figure S11, Supporting Information), and it turned out that BDP‐2 NPs produced ^1^O_2_ in the dark. By contrast, due to the lack of anthracenyl groups, no ^1^O_2_‐generation was observed for both BDP NPs and NBDP NPs (Figure S11, Supporting Information), which demonstrated their poor ^1^O_2_‐generation capability in the dark state.

Encouraged by the excellent ^1^O_2_‐release capability of ANBDP NPs in the dark‐and‐hypoxia state, we further investigated the cancer cell killing effect of ANBDP NPs via methyl thiazolyl‐tetrazolium (MTT) assays. As shown in Figure S12a (Supporting Information), only 730 nm light irradiation would cause no toxicity to 4T1 cells. Without light‐irradiation, ANBDP NPs presented little toxicity to 4T1 cells (Figure 3e). However, once triggered with 730 nm irradiation, ANBDP NPs exhibited a strong inhibition effect on the growth of 4T1 cells with half‐maximal inhibitory concentration (IC_50_) of 6.7 µg mL^–1^ (Figure 3e). For groups pretreated with CQ, ANBDP NPs caused weak toxicity to 4T1 cells, with 74% viability incubated with 100 µg mL^–1^ of ANBDP NPs. This demonstrated that the protonation process in endosomes greatly contributed to the ^1^O_2_‐photogeneration capability of ANBDP NPs. Furthermore, the cancer cell killing effect of ANBDP NPs under hypoxia was also investigated. As shown in Figure 3f, ANBDP NPs presented an excellent PDT efficacy in the hypoxia state (IC_50_ = 7.2 µg mL^−1^), which was comparable to that in the normoxia state (IC_50_ = 6.7 µg mL^−1^). In contrast, due to the lack of special functional groups, BDP NPs exhibited poor phototherapeutic efficacy under both 21% O_2_ and 2% O_2_ (Figure S12b, Supporting Information). Although NBDP NPs presented good phototoxicity (IC_50_ = 19.6 µg mL^−1^) under normoxia, its photodynamic activity under hypoxia was relatively weak (Figure S12c, Supporting Information). At a dosage of 100 µg mL^−1^, NBDP NPs exhibited 79.2% cell viability, denoting the inferiority of NBDP NPs over ANBDP NPs for killing hypoxic cancer cells. Moreover, both cancer cells (4T1) and normal cells (LO2, HUVECs, and HaCaT) treated with ANBDP NPs without photoirradiation exhibited high cell viability even at 100 µg mL^−1^ (Figure S12d, Supporting Information), manifesting the negligible dark toxicity and excellent biocompatibility of ANBDP NPs.

To visually convince the cytotoxicity of ANBDP NPs, 4T1 cells with different treatment was further evaluated by Calcein‐AM (green fluorescence for live cells) and propidium iodide (PI, red fluorescence for dead cells) staining assays (Figure 3g). At the concentration of 100 µg mL^−1^, almost no cells were killed by ANBDP NPs without irradiation. For cells pretreated with CQ, only a few 4T1 cells were killed by ANBDP NPs owing to the increase of endosomal pH, which was unfavorable for the activation of ANBDP photosensitizers to the CT off state. Moreover, under both normoxia and hypoxia states, most cells were killed by ANBDP NPs, which further confirmed the significant cancer‐killing effect of ANBDP NPs with hypoxia tolerance.

Fluorescence cytometry assays were carried out to investigate the cancer‐killing manner in detail. As shown in Figure 3h, 4T1 cells showed a 97.0% survival rate without 730 nm irradiation, and no early‐stage or late‐stage apoptosis was observed. While for 4T1 cells incubated with 100 µg mL^–1^ of ANBDP NPs with irradiation, the survival rate of 4T1 cells drastically decreased to 15.8%. Meanwhile, 65.3% late‐stage apoptosis and 10.3% early‐stage were observed for 4T1 cells, confirming the cytotoxicity of ANBDP NPs. Considering that the B‐cell lymphoma‐2 (Bcl‐2) family proteins as the master regulator of genes participate in cell apoptosis, we thus employed the western blotting (WB) technique to examine expressions of Bcl‐2 (anti‐apoptotic protein) and Bax (pro‐apoptotic protein) in 4T1 cells treated with ANBDP NPs. As shown in Figure 3i, cells treated with ANBDP NPs were witnessed with downregulation of Bcl‐2 expression and upregulation of Bax expression. Moreover, the PDT effect of ANBDP NPs also promoted the overexpression of apoptosis implementing gene caspase‐3. These results confirmed that ANBDP NPs could induce the apoptosis of hypoxic tumor cells via dark and hypoxia‐tolerant PDT.

**Figure 4:**
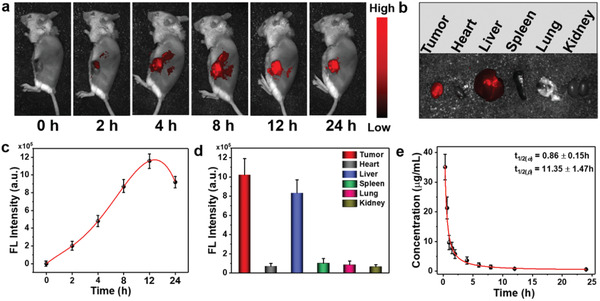
a) In vivo fluorescence imaging of ANBDP NPs in 4T1 tumor‐bearing mice at different time points. b) Fluorescence imaging of ANBDP NPs distributed in tumor and normal organs (heart, liver, spleen, lung, and kidney). c) Fluorescence intensity changes of ANBDP NPs in (a) (*n* = 3, mean ± SD). d) Fluorescence intensity change of ANBDP NPs in normal organs and tumor tissues in Figure 4b (*n* = 3, mean ± SD). e) Pharmacokinetic profiles of mice injected with ANBDP NPs (*n* = 3, mean ± SD).

To further investigate in vivo performance of ANBDP NPs, a fluorescence imaging system was employed to assess the accumulation kinetics of ANBDP NPs in 4T1 tumors. As shown in **Figure** **4**a, the fluorescence of ANBDP NPs in tumor tissues increased and reached the maximum at 12 h post‐injection (Figure 4c). After that, the fluorescence in tumor tissues began to decrease, indicating a reasonable degradation rate of ANBDP NPs. To investigate the biodistribution of ANBDP NPs, tumor tissues and normal tissues (heart, liver, spleen, lung, and kidney) were taken out for fluorescence imaging at 24 h post‐injection. It was found that robust fluorescence distributed in the tumors, suggesting selective accumulation of ANBDP NPs in tumor tissues (Figure 4b,d). Considerable fluorescence could be observed in livers, manifesting that ANBDP NPs could be scavenged by hepatic metabolism. Meanwhile, negligible fluorescence presented in the heart, spleen, lung, and kidney, indicating the excellent biosafety of ANBDP NPs.

Furthermore, NIR fluorescence of ANBDP NPs in blood was tracked by a spectrometer to understand the in vivo pharmacokinetic manner of ANBDP NPs. Blood of tumor‐bearing mice (injected with a saline solution containing ANBDP NPs) was collected at different time points and measured immediately. As shown in Figure 4e, the calculated pharmacokinetic curves of ANBDP NPs were well complied with a two‐compartment model with the blood half‐life of *t*_1/2(_ *_*α*_*_)_ = 0.86 ± 0.15 h and *t*_1/2(_ *_*β*_*_)_ = 11.35 ± 1.47 h. We also found that the concentration of ANBDP NPs decreased quickly in a time‐dependent manner. Almost no fluorescence could be detected at 24 h post‐injection, which indicated that ANBDP NPs could be rapidly metabolized through blood circulation.

**Figure 5:**
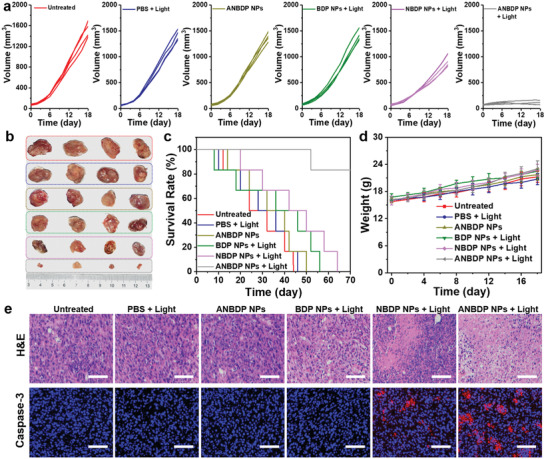
a) Volume changes of 4T1 tumors receiving different treatment. b) Photographs of tumors taken from mice after 18 days of treatment, red frame: untreated; blue frame: PBS + light; dark yellow frame: ANBDP NPs without light irradiation; green frame: BDP NPs + light; pink frame: NBDP NPs + Light; gray frame: ANBDP NPs + Light. c) Survival rate of mice in different groups after receiving 18 days of treatment. d) Body weight changes of mice in different groups. e) H&E staining and caspase‐3 staining of tumor tissues. Scale bars: 50 µm.

Encouraged by unique properties of ANBDP NPs involving proton‐driven transformation, endosomal escape, and dark‐and‐hypoxia tolerant ^1^O_2_‐generation, we evaluated the antitumor efficacy of ANBDP NPs on 4T1‐tumor bearing BALB/c mice models. To quantitatively assess the therapeutic efficacy, tumor growth rates of ANBDP NPs‐, NBDP NPs‐, or BDP NPs‐treated mice were continuously recorded for 18 days after 730 nm laser irradiation. Without laser irradiation, the tumor volumes of ANBDP NPs‐treated mice were similar to that of untreated and saline‐treated groups (**Figure** **5**a). BDP NPs with laser irradiation did not provide any significant tumor growth inhibition effect compared with the untreated group. For mice injected with NBDP NPs receiving irradiation, tumor growth was inhibited to a certain degree (53.2% inhibition rate) due to the photogeneration of ^1^O_2_ under an acidic environment. Distinctly, tumor growth of ANBDP NPs treated mice with irradiation was effectively suppressed (96.7% inhibition rate) compared to that of untreated mice, demonstrating the excellent antitumor efficacy of ANBDP NPs. The representative photographs after treatment (Figure 5b; Figure S13 in Supporting Information) also indicated the superior antitumor effect of ANBDP NPs over NBDP NPs and BDP NPs. As such, ANBDP NPs treated mice with irradiation exhibited the longest life span (83.3% survival rate in 70 days) than other groups of mice (Figure 5c), which further clarified the superiority of ANBDP NPs over BDP NPs and NBDP NPs for antitumor treatment. Meanwhile, as shown in Figure 5d, no abnormal body weight loss was found during 18 days of treatment, suggesting the excellent biosafety of ANBDP NPs.

Next, the potential toxicology of ANBDP NPs to normal organs and tumor tissues was further investigated by histological examinations. Normal tissues, including heart, kidney, lung, liver, and spleen, were collected for hematoxylin–eosin (H&E) staining analysis after 18 days of treatment. As shown in Figure S14 (Supporting Information), there was no abnormal morphological change on normal organs of all treatment groups in comparison with healthy mice, implying the favorable biosafety of these therapeutic agents. To further investigate the antitumor efficacy of ANBDP NPs, the tumors of all groups were extracted and sectioned for histological analysis utilizing H&E staining and caspase‐3 immunofluorescence staining. As demonstrated by H&E staining results in Figure 5e and Figure S15 (Supporting Information), no obvious histological damages could be observed in the untreated group, PBS + light group, ANBDP NPs group, and BDP NPs + light group, suggesting that no toxicity to tumors was caused by these treatments. However, the tumor slices of ANBDP NPs‐treated mice with 730 nm light irradiation exhibited extensive nuclear shrinkage and disappearance area, much larger than that of NBDP NPs‐treated mice, indicating that ANBDP NPs showed stronger phototoxicity to 4T1 cancer cells than NBDP NPs. In addition, caspase‐3 immunofluorescence images revealed that no obvious apoptosis in tumor regions was found for untreated, PBS + light, ANBDP NPs, or BDP NPs + light‐treated mice. In comparison, prominent red fluorescent signals of caspase‐3 expression were observed for ANBDP NPs‐treated mice with 730 nm laser irradiation, which was much stronger than that of NBDP NPs‐treated mice. All these results illustrated the superior therapeutic efficacy of ANBDP NPs over NBDP NPs and BDPs, which was in high consistency with the in vivo treatment outcomes.

The potential long‐term systemic biotoxicity of ANBDP NPs was assessed by collecting blood and serum from healthy mice and treated mice. Hematological indexes, such as white blood cells (WBC), red blood cells (RBC), mean corpuscular volume (MCV), mean corpuscular hemoglobin (MCH), mean corpuscular hemoglobin concentration (MCHC), hematocrit (HCT), platelets (PLT), and hemoglobin (HGB), were examined at different days after administration of ANBDP NPs (Figure S16, Supporting Information). Compared with the blank group (healthy mice), no abnormal changes were detected during 2 weeks in mice treated with ANBDP NPs, suggesting that ANBDP NPs would not cause any infection and inflammation in vivo. Moreover, serum hepatic–renal function indexes, such as alanine aminotransferase (ALT), aspartate aminotransferase (AST), total protein (TP), albumin/globulin (A/G), creatinine (CREA), urea (UREA), globulin (GLOB), and albumin (ALB) were detected and showed that no systemic side effects would be induced by ANBDP NPs in 14 days (Figure S17, Supporting Information). Taken together, these indexes indicated that ANBDP NPs would not cause long‐term systemic biotoxicity.

## 3 Conclusion

In summary, proton‐driven transformable ^1^O_2_‐nanotrap ANBDP NPs are prepared to improve the PDT efficiency. Triggered by a proton‐rich endosomal environment, the protonated ANBDP NPs could escape from endosomal entrapment via “proton‐sponge”‐like effect, avoiding the recruitment and digestion function of degradation enzymes. Meanwhile, the proton‐rich endosomal environment also promoted the morphology‐and‐size transformation of^1^O_2_‐nanotrp from nanocubes (94.1 nm in length) to nanospheres (12.3 nm in diameter), where the ^1^O_2_‐photogeneration performance gave a 15.8‐fold amplification. Upon excitation with a 730 nm laser, ANBDPH encapsulated in the ^1^O_2_‐nanotrap significantly sensitized ^3^O_2_ for ^1^O_2_ photogeneration and simultaneously captured as‐generated ^1^O_2_ to form the endoperoxide ANOBDPH, allowing later‐stage sustainable ^1^O_2_ release in the dark and hypoxia state. As such, ANBDP NPs achieved a 96.7% suppression rate of tumor growth, much higher than that of NBDP NPs and BDP NPs. Beneficial from proton‐driven transformation, endosomal escape, ^1^O_2_‐release in dark‐and‐hypoxia, and biosafety, the ^1^O_2_‐nanotrap ANBDP NPs hold great promise for cancer phototherapy. Thus, this study provides a new paradigm to design transformable therapeutic nanoagents for enhanced cancer PDT.

## 4 Experimental Section

### Materials and Characterizations

DSPE‐mPEG_2000_ was purchased from Shanghai Yare Co. Ltd. ROS probes, TEMPO, DMPO, SOSG, and DCFH‐DA, were purchased from Adamas‐Beta. Hypoxia probe ROS‐ID was purchased from Enzo Life Sciences Co. Ltd. (USA). All other chemical agents were purchased from Shanghai Titan Scientific Co. Ltd. Nuclear Magnetic Resonance (NMR) spectra were measured utilizing JEOL ECZ‐400 spectrometer (400 MHz) and Bruker Ultra Shield Plus (400 MHz). Mass spectroscopy of photosensitizers was measured using MALDI‐TOF mass instruments. The diameter of nanoparticles was characterized by a dynamic light scattering particle size analyzer (NanoPlus, Micromeritics Instrument Co. Ltd.). UV–vis absorption was measured by a UV‐3600 Shimadzu UV–vis–NIR spectrometer. Fluorescence spectra were tested with a Thermo Fisher fluorophotometer, and the fluorescence decay was obtained on Edinburgh FLS 1000 instrument. Confocal fluorescence imaging was carried out employing Olympus IX 70 imaging systems. In vivo fluorescence images of tumor‐bearing mice were conducted by using Fluor Vivo 2000 INDEC imaging system.

### Preparation of Therapeutic Nanoagents

Under sonication environment (250 W), 1.5 mg ANBDP (2 mmol) in 10 mL THF was swiftly added into 10 mL water solution, containing 10 mg DSPE‐mPEG_2000_. THF was evaporated under reduced pressure after 5 min of sonication. Thereafter, a green aqueous solution was obtained, and it was further centrifuged and filtered via a 220 nm filter to obtain the ANBDP NPs for further application. The preparation methods for BDP NPs, BDP‐2 NPs, and NBDP NPs are similar to ANBDP NPs.

### ROS Detection

ROS generation was characterized using the electron spin resonance (ESR) method and the fluorescent method. To qualitatively detect the type of ROS, DMPO and TEMPO were employed as ESR probes to detect radicals and singlet oxygen, relatively. ANBDP NPs (50 µg mL^–1^) and DMPO (5 mg mL^–1^) were dissolved in water receiving irradiation of 730 nm laser (0.05 W cm^–2^) for 30 s. The product was immediately tracked with an EPR spectrometer to determine the generated O_2_^●−^ or OH^●^. Furthermore, SOSG was employed as ^1^O_2_ specific fluorescence indicator to quantitatively measure ^1^O_2_ generation. To quantitatively detect ^1^O_2_, the fluorescence of mixture containing 0.01 × 10^‐3^ m SOSG and 20 µg mL^−1^ ANBDP NPs were recorded using a fluorescent spectrometer each time after 730 nm laser irradiation (0.05 W cm^−2^). The ^1^O_2_ generation of BDP NPs and NBDP NPs was detected in the same method with ANBDP NPs.

### Cell Culture and Measurements

4T1 cells, HaCaT cells, and LO2 cells were obtained from the School of Pharmaceutical Science, Nanjing Tech University. Human umbilical vein endothelial cells (HUVECs) were purchased from Wuhan Servicebio Co. Ltd. 4T1 cells and LO2 cells were cultured in 1640 medium containing fetal bovine serum (FBS, 10%, v/v) and antibiotics (1%, v/v) at 37 °C and 5% CO_2_ atmosphere. HUVECs were cultured in a HUVEC‐specific medium at 37 °C and 5% CO_2_ atmosphere. HaCaT cells were cultured in Dulbecco's modified Eagle's medium (Gibco), containing FBS (10%, v/v) and antibiotics (1%, v/v) under a humidified atmosphere of 5% CO_2_ at 37 °C. An anaerobic jar was employed for hypoxic cell culture. To investigate subcellular distributions, 4T1 cells (2 × 10^5^) were seeded in glass‐bottom Petri dishes that contain 1640 medium cultured for 24 h. Then 6.7 µg mL^–1^ of nanoparticles were added and further cultured for 4 and 12 h, respectively. The 1640 culture medium was removed, and LysoTracker green was added and incubated for 30 min. The dishes were washed three times. The fluorescence of ANBDP NPs was detected utilizing a laser confocal fluorescence microscope excited at 633 nm. The fluorescence of the LysoTracker was detected utilizing a laser confocal fluorescence microscope excited at 488 nm. Cellular uptake of BDP NPs and NBDP NPs was also treated similarly with ANBDP NPs. To detect ROS Generation in cells: In a glass‐bottom petri dish, 2 × 10^5^ 4T1 cells were seeded and incubated with 1640 medium for 24 h. Then, 6.7 µg mL^–1^ of the solution was added and cultured for 12 h. To detect ^1^O_2_‐photogeneration, Thereafter, DCFH‐DA (10 µg mL^–1^) were added and incubated for 30 min. Thereafter, 4T1 cells incubated with ANBDP NPs received 60 s irradiation. Next, free DCFH‐DA was removed, and stained cells were washed to be measured with a confocal fluorescence microscope excited at 488 nm. To detect ^1^O_2_ generated in the dark state, 4T1 cells incubated with ANBDP NPs received 60 s irradiation. Thereafter, DCFH‐DA (10 µg mL^−1^) were added and incubated for 30 min. In the following, free DCFH‐DA was removed, and stained cells were washed to be measured with a confocal fluorescence microscope excited at 488 nm. The hypoxic level in cells was detected in the same way using ROS‐ID excited at 633 nm. To measure the cytotoxicity of nano agents, MTT assays were carried out. A total of 5 × 10^3^ cells per well were seeded in 96‐well plates cultured for 24 h. Then, nanoagents were added at different concentrations (0, 1, 2, 5, 10, 20, 50, 100 µg mL^−1^) and cultured for 24 h. Next, 20 µL of MTT (5 mg mL^−1^) was added to each well of the plates and further incubated for 4 h. Thereafter, the culture medium was removed, and 200 µL dimethyl sulfoxide was added to dissolve the generated formazan. The absorption of DMSO solution was measured using an enzyme‐labeled instrument.

### Animal Model

Four‐week‐old female athymic BALB/c mice (Permit number: SCXK(Su)201 7‐0001) were purchased from Nanjing Qinglongshan Animal Reproduction Center. All animal experiments were approved and guided by the School of Pharmaceutical Science, Nanjing Tech University, in compliance with relevant laws and guidelines. 4T1 cells were inoculated in the left subcutaneous flank of BALB/c mice. When the volume of 4T1 tumors reached 50–100 mm^3^, mice were randomly divided for different treatments.

### In Vivo Tumor Imaging

To real‐time detect the tumor‐targeting ability of ANBDP NPs, 4T1‐tumor bearing mice were injected with the solution of ANBDP NPs (100 µL, 100 µg mL^−1^). Then the fluorescence of ANBDP NPs was measured at a different time (0, 2, 4, 8, 12, 24 h) with Fluor Vivo 2000 INDEC bioimaging system. At 24 h, normal organs (heart, liver, spleen, lung, and kidney) and tumor tissues were taken out for fluorescence imaging to study the biodistribution of ANBDP NPs.

### In Vivo Treatment

To investigate in vivo therapeutic efficacy of ANBDP NPs, tumor‐bearing balb/c mice were randomly divided into the following six groups for different treatments: i) Untreated, ii) PBS + Light, iii) ANBDP NPs, iv) BDP NPs + Light, v) NBDP NPs + Light, and vi) ANBDP NPs + Light. For groups with light irradiation, mice received 730 nm laser irradiation of 0.05 W cm^‐2^ for 5 min every 2 days. Furthermore, tumor volumes and body weight were also measured every 2 days. Tumor volumes were calculated according to the equation: volume = length × (width)^2^/2.

### Histological Examination

After 18 days of treatment, mice were sacrificed for histological examination. Normal tissues Heart, lung, kidney, spleen, livers, and 4T1 tumor tissues were taken out to prepare paraffin‐fixed slices. These slices were conducted with H&E and DHE staining assay for further histological analysis.

### Blood Routine and Hepatic‐Renal Function Examination

Blood routine examinations were performed by collecting the blood at a different time point after injection of ANBDP NPs. Functional markers WBC, RBC, HGB, HCT, MCV, MCH, MCHC, and PLT, were measured by Wuhan Servicebio Co. Ltd. Hepatorenal Function Examination were performed by collecting the blood serums at a different time point after injection of ANBDP NPs. Functional markers TP, A/G, ALT, AST, GLOB, AST, UREA, CREA, and GGT were measured by Wuhan Servicebio Co. Ltd.

### Statistical Analysis

Normalization was applied for data processing. Statistical analysis was conducted by one‐way analysis of variance and student's *t*‐test. *p* < 0.05 was considered statistically significant. Sample size (*n*) and mean ± standard deviations were utilized for results expression.

## Conflict of Interest

The authors declare no conflict of interest.

## Supporting information

Supporting Information

## Data Availability

The data that support the findings of this study are available from the corresponding author upon reasonable request.
