# Supplementary material for: Proton‐Driven Transformable 1O2‐Nanotrap for Dark and Hypoxia Tolerant Photodynamic Therapy
Source: Adv Sci (Weinh). 2022 Apr 18;9(17):2200128. doi: 10.1002/advs.202200128 (PMC9189669; doi:10.1002/advs.202200128)
Supplement: Supplementary file 1 — Supporting Information [file ADVS-9-2200128-s001.pdf]

ADVANCED SCIENCE
Open Access

Supporting Information

for *Adv. Sci.*, DOI 10.1002/advs.202200128

Proton-Driven Transformable $^1O_2$-Nanotrap for Dark and Hypoxia Tolerant Photodynamic Therapy

Dapeng Chen, Hanming Dai, Weili Wang, Yu Cai, Xiaozhou Mou, Jianhua Zou, Jinjun Shao*, Zhengwei Mao, Liping Zhong, Xiaochen Dong* and Yongxiang Zhao**

Supporting Information

Proton-Driven Transformable $^{1}O_2$-Nanotrap for Dark and Hypoxia Tolerant Photodynamic Therapy

Dapeng Chen, Hanming Dai, Weili Wang, Yu Cai, Xiaozhou Mou, Jianhua Zou, Jinjun Shao,* Zhengwei Mao, Liping Zhong, Xiaochen Dong,* Yongxiang Zhao**

1. Synthesis of Photosensitizers

Synthesis of BDP-1.

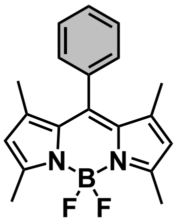

BDP-1

0.64 g of Benzaldehyde (6.0 mmol) and 1.24 g of 2,4-dimethylpyrrole (13.2 mmol) were added in a round-bottom flask and dissolved in 180 mL of tetrahydrofuran (THF). 1.0 mL CF_3COOH was added in THF solution, which was kept stirring for 12 h at room temperature. After that, 240 mL fresh THF containing 2,3-dicyano-5,6-dichlorobenzoquinone (DDQ, 1.36 g) was added into the reaction mixture and stirred for another four hours. Thereafter, 36 mL triethylamine (TEA) and 36 mL BF_3•OEt_2 were added into the solution at an ice bath for reaction overnight. Then THF was evaporated, and the residues were purified with chromatography on silica gel (CH_2Cl_2/petroleum ether (PE) = 1:1) to obtain the orange product (0.93 g, 48%). ^{1}H NMR (400 MHz, $CDCl_3$): δ ppm 7.48 (t, J = 1.2 Hz, 1H, Ar-H), 7.47 (d, J = 1.6 Hz, 2H, Ar-H), 7.28 (dd, J = 2.4 Hz, 7.2 Hz, 2H, Ar-H), 5.97 (s, 2H, pyrrole-H), 2.54 (s, 6H, Ar-CH_3), 1.36 (s, 6H, Ar-CH_3).

Synthesis of BDP.

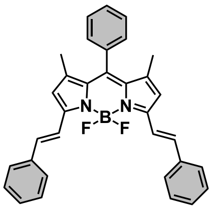

BDP

BDP-1 (0.65 g, 2.0 mmol) and benzaldehyde (0.49 g, 4.8 mmol) was dissolved in 40 mL *N*,*N*-dimethylformamide (DMF). 1 mL piperidine and 1 mL CH_3COOH were added with

reaction at 120 °C for 3 h. 500 mL of purified H_2O was added into the crude product followed with extraction using CH_2Cl_2. Finally, the crude product was purified with chromatography on silica gel (ethyl acetate (EA)/PE = 1:3) to obtain dark brwon solid (0.46 g, 46%). ^{1}H NMR (400 MHz, $CDCl_3$): δ ppm 7.72 (d, J = 8.4 Hz, 2H, -C=CH-), 7.62 (d, J = 7.6 Hz, 4H, Ar-H), 7.50 (t, J = 2.4 Hz, 1H, Ar-H), 7.49 (d, J = 2.0 Hz, 2H, -C=CH-), 7.40 (t, J = 7.2 Hz, 4H, Ar-H), 7.31 (dd, J = 2.0 Hz, 7.2 Hz, 4H, Ar-H), 7.27 (dd, J = 2.4 Hz, 16.4 Hz, 2H, Pyrrole-H), 6.64 (s, 2H, pyrrole-H), 1.44 (s, 6H, Ar-H).

Synthesis of NBDP.

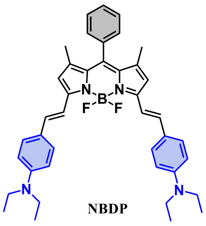

NBDP

BDP-1 (0.65 g, 2.0 mmol) and 4-diethylamino-benzaldehyde (0.85 g, 4.8 mmol) was dissolved in 40 mL DMF. 1 mL piperidine and 1 mL CH_3COOH were added with reaction at 120 °C for 3 h. 500 mL purified H_2O was added into the crude product followed with extraction using CH_2Cl_2. Finally, the crude product was purified with chromatography on silica gel (EA/PE = 1:2) to obtain green solid (0.53 g, 41%). ^{1}H NMR (400 MHz, $CDCl_3$): δ ppm 7.52 (d, J = 8.8 Hz, 4H, Ar-H), 7.46 (t, J = 3.6 Hz, 2H, Ar-H), 7.45 (d, J = 2.0 Hz, 2H, -C=CH-) 7.32 (d, J = 2.8 Hz, 2H, Ar-H), 7.31 (d, J = 1.6 Hz, 1H, Ar-H) 7.18 (d, J = 14.8 Hz, 2H, -C=CH-), 6.67 (d, J = 8.8 Hz, 4H, Ar-H), 6.58 (s, 2H, pyrrole-H), 3.41 (d, J = 6.4 Hz, 8H, -N-CH_2-), 1.40 (s, 6H, Ar-CH_3), 1.19 (s, 12H, -C-CH_3).

Synthesis of BDP-2.

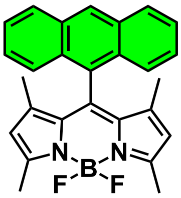

BDP-2

1.24 g of anthracene-9-carbaldehyde (6.0 mmol) and 1.24 g of 2,4-dimethylpyrrole (13.2 mmol) were added in a round-bottom flask and dissolved in 180 mL tetrahydrofuran (THF). 1.0 mL CF_3COOH was added in THF solution, which was kept stirring for 12 h at room temperature. Thereafter, 240 mL anhydrous THF containing 2,3-dicyano-5,6-dichloro-benzoquinone (DDQ, 1.36 g) was added into the reaction mixture and stirred for another 4 h. Thereafter, 36 mL of triethylamine (TEA) and 36 mL BF_3•OEt_2 were added into the solution at an ice bath for reaction overnight. Then THF was evaporated, and the residues were purified with chromatography on silica gel (CH_2Cl_2/PE = 1:1) to obtain the dark-red solid (1.07 g, 42%). ^{1}H NMR (400 MHz, $CDCl_3$): δ ppm 8.56 (s, 1H, Ar-H), 8.01 (d, J = 8.4 Hz, 2H, Ar-H), 7.48 (dd, J = 6.4 Hz, 14.4 Hz, 2H, Ar-H), 7.41 (dd, J = 5.2 Hz, 14.0 Hz, 2H, Ar-H), 7.26 (d, J= 2.8 Hz, 2H, Ar-H), 5.88 (s, 2H, pyrrole-H), 5.97 (s, 2H, pyrrole-H), 2.54 (s, 6H, Ar-CH_3), 1.36 (s, 6H, Ar-CH_3).

Synthesis of ANBDP.

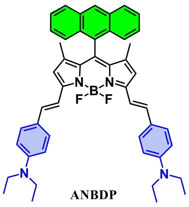

BDP-2 (0.85 g, 2.0 mmol) and 4-diethylamino-benzaldehyde (0.85 g, 4.8 mmol) was dissolved in 40 mL DMF. 1 mL piperidine and 1 mL of CH_3COOH were added with reaction

at 120 °C for 3 h. 500 mL of H_2O was added into the product followed with extraction using CH_2Cl_2. Finally, crude product was purified with chromatography on silica gel (EA/PE = 2:3) to obtain dark-green solid (0.55 g, 37%). ^1H NMR (400 MHz, $CDCl_3$): δ ppm 8.57 (s, 1H, Ar-H), 8.02 (dd, J = 8.4 Hz, 21.6 Hz, 4H, Ar-H), 7.62 (d, J = 16.0 Hz, 2H, -C=CH-), 7.55 (d, J = 8.8 Hz, 4H, Ar-H), 7.49 (t, J = 6.8 Hz, 2H, Ar-H), 7.41 (t, J = 7.2 Hz, 2H, Ar-H), 7.20 (d, J 16.0 Hz, 2H, -C=CH-) 6.70 (d, J = 8.8 Hz, 4H, Ar-H), 6.51 (s, 2H, pyrrole-H), 3.43 (d, J = 7.2 Hz, 8H, -N-CH_2-), 1.58 (s, 6H, Ar-CH_3), 1.21 (s, 12H, -C-CH_3). ^{13}C NMR (100 MHz, $CDCl_3$): 153.03, 148.34, 140.43, 136.55, 133.91, 131.29, 130.44, 129.37, 129.26, 128.20, 127.86, 126.66, 125.70, 125.62, 124.38, 121.90, 117.07, 114.48, 111.51, 44.48, 13.44, 12.66. MALDI-TOF (m/z): calcd for $[C_{49}H_{49}BF_2N_4]^+$: 742.7658; found, 742.4725.

2. Supporting Figures

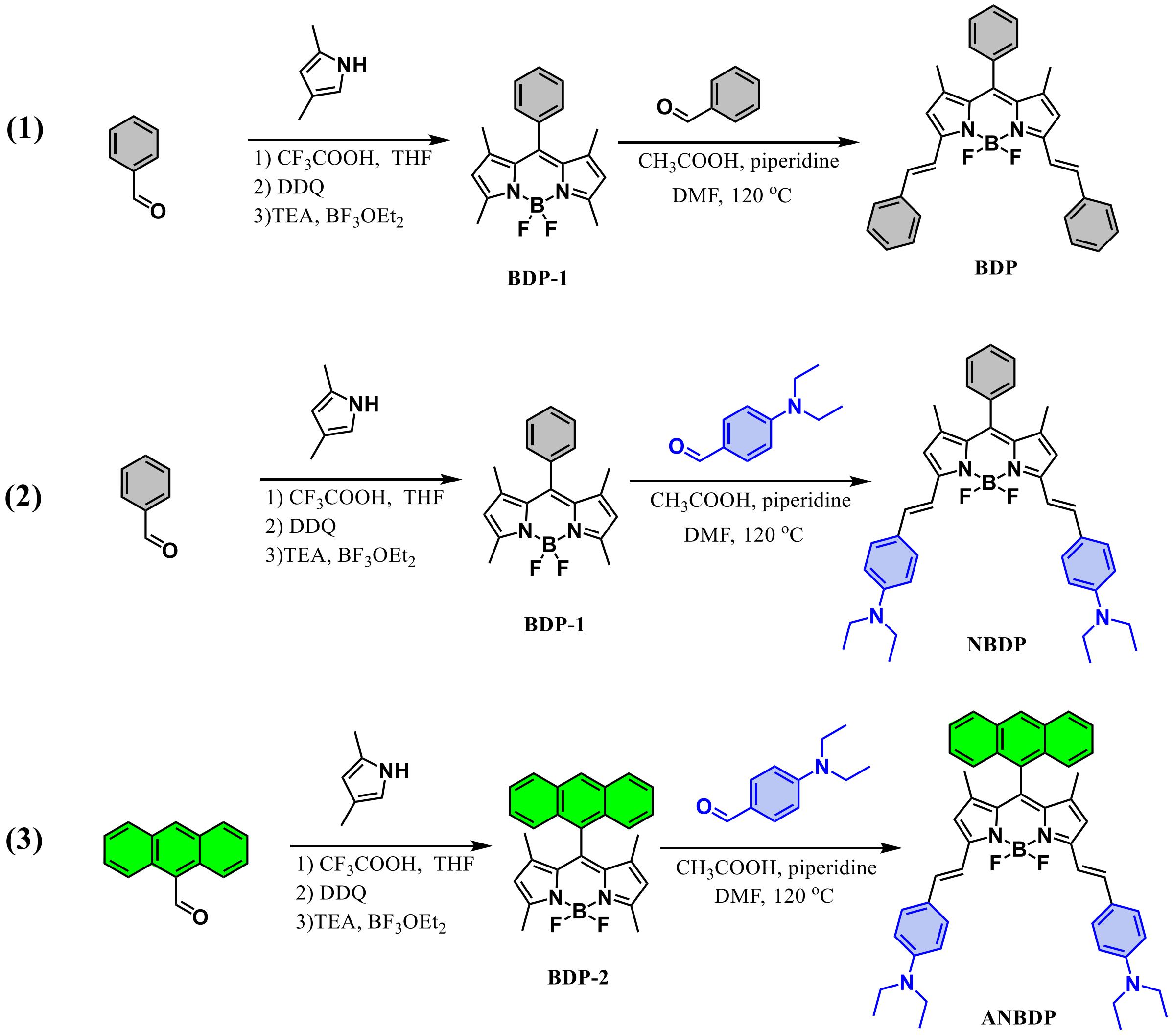

Figure S1. Synthetic routes for BDP, NBDP, and ANBDP.

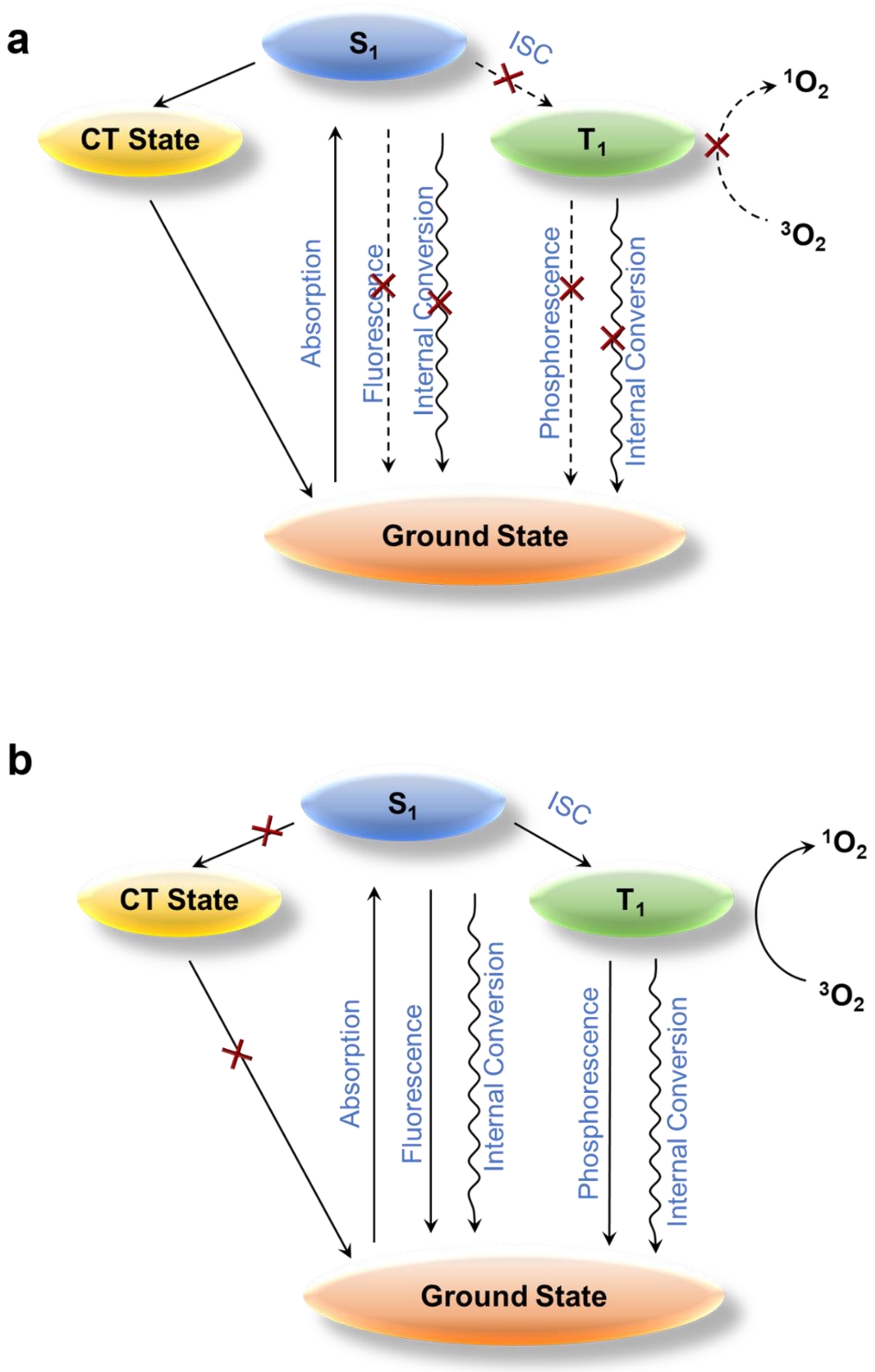

Figure S2. a) Proposed charge-transfer (CT) state of ANBDP. b) Proposed CT state off mechanism of ANBDPH for fluorescence emission and $^1O_2$ generation.

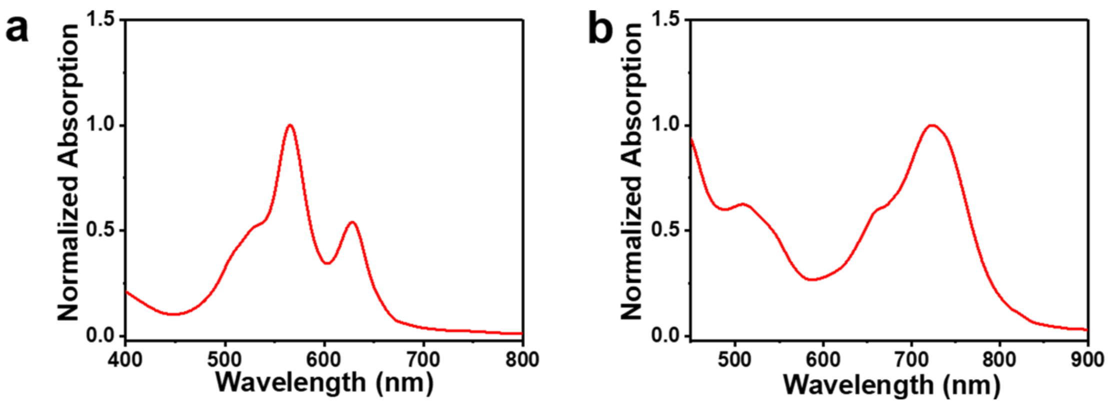

Figure S3. a) Normalized absorption of BDP NPs. b) Normalized absorption of NBDP NPs.

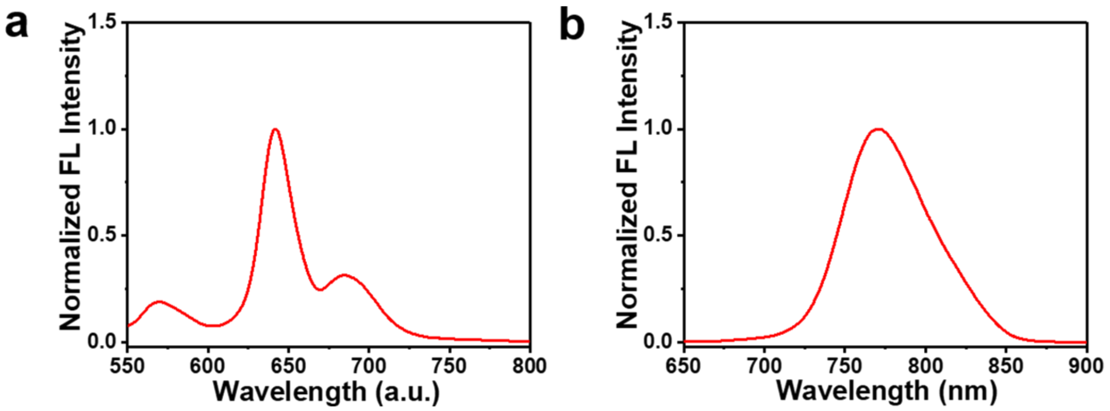

Figure S4. Fluorescence (FL) spectra for a) BDP NPs, b) NBDP NPs.

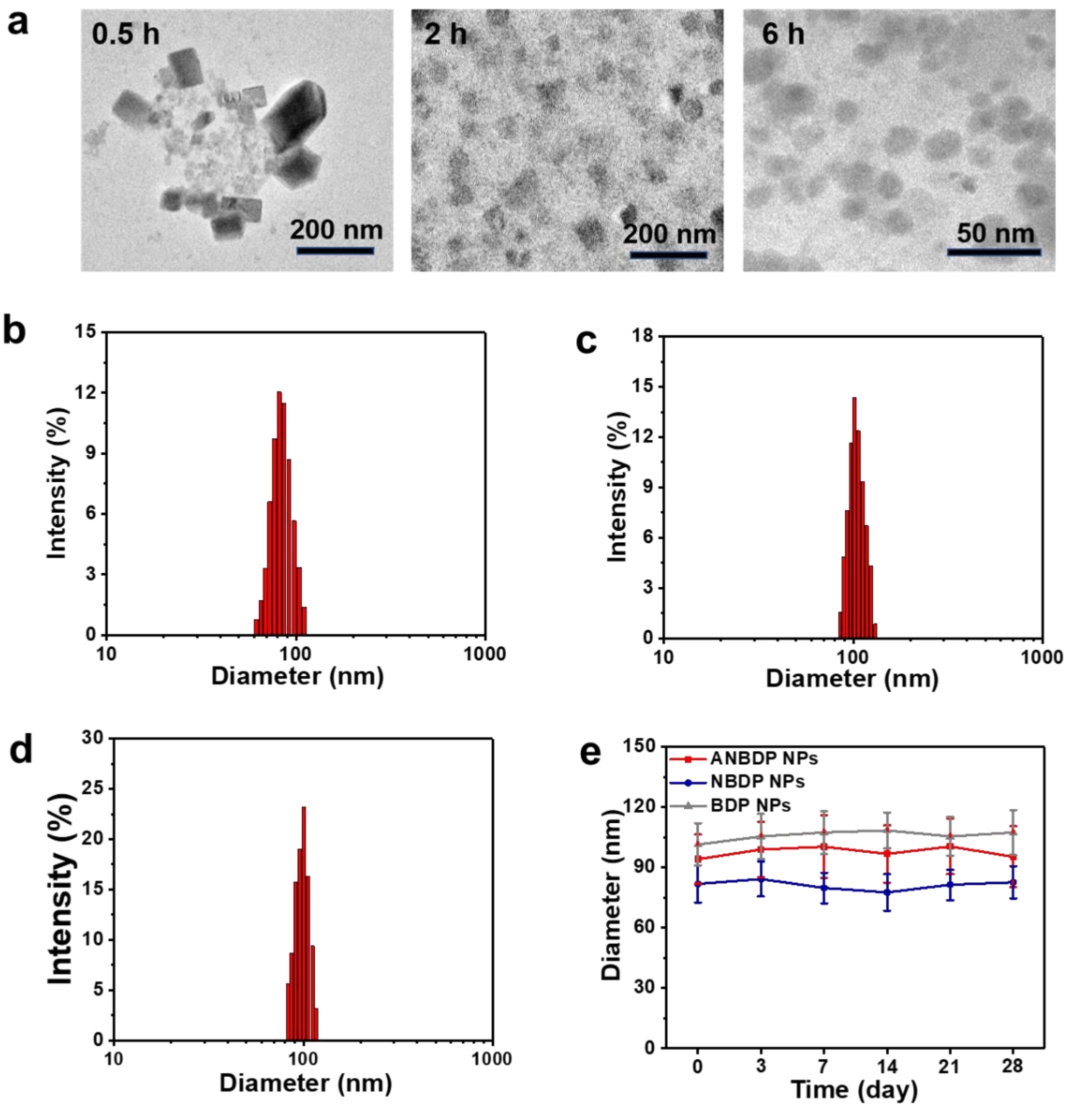

Figure S5. a) TEM images of ANBDP NPs at different times after the medium changes from pH 7.4 to pH 5.0. b) Size distribution of NBDP NPs. c) Size distribution of BDP NPs. d) Size distribution of BDP-2 NPs. e) Long-term stability of ANBDP NPs, NBDP NPs, and BDP NPs.

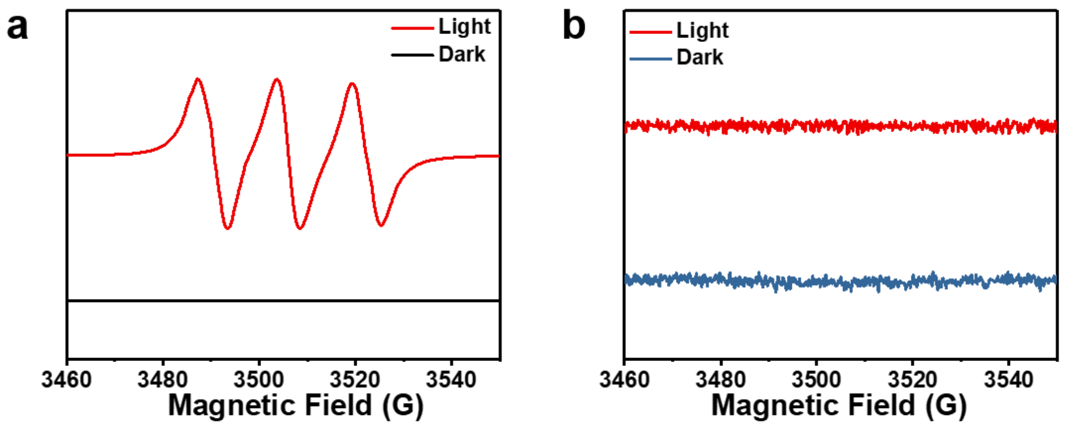

Figure S6. a) $^1O_2$ generation of ANBDP NPs (at pH 5.0) detected by TEMPO under light (730 nm, 0.05 W cm^{-2}) or dark. b) $HO^{\bullet}$ and $O_2^{\bullet -}$ generation of ANBDP NPs (pH 5.0) detected by DMPO under light (730 nm, 0.05 W cm^{-2}) or dark.

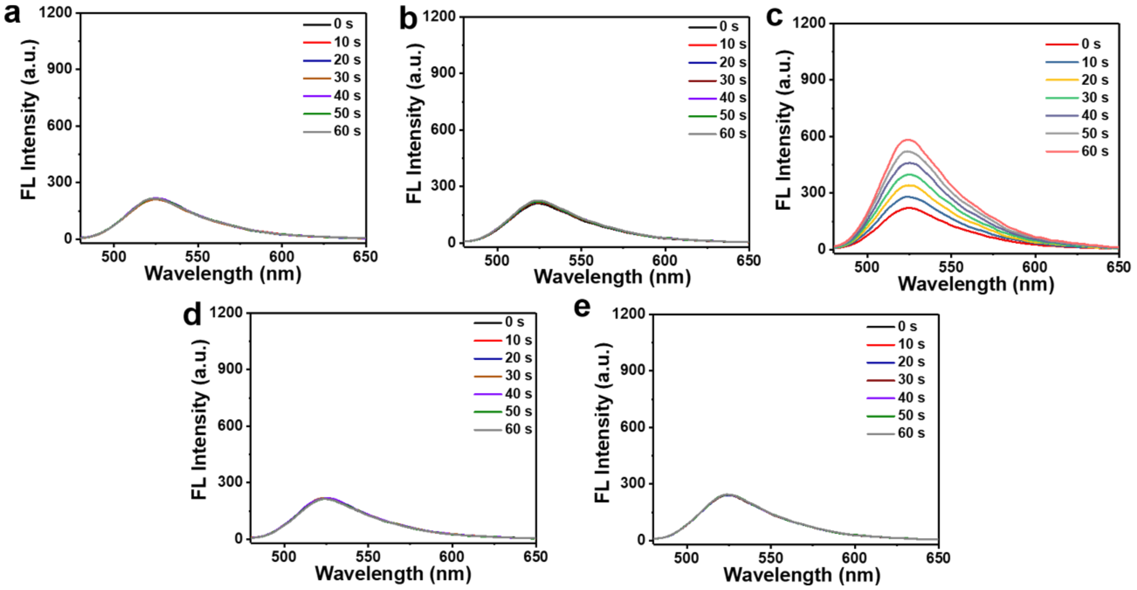

Figure S7. a) Fluorescence intensity change of SOSG triggered by ANBDP NPs under dark. b, c) Fluorescence intensity change of SOSG triggered by NBDP NPs at pH 7.4 and 5.0, respectively, under irradiation. d, e) Fluorescence intensity change of SOSG triggered by BDP NPs at pH 7.4 and 5.0, respectively, under irradiation. Laser: 730 nm, 0.05 Wcm^{-2}.

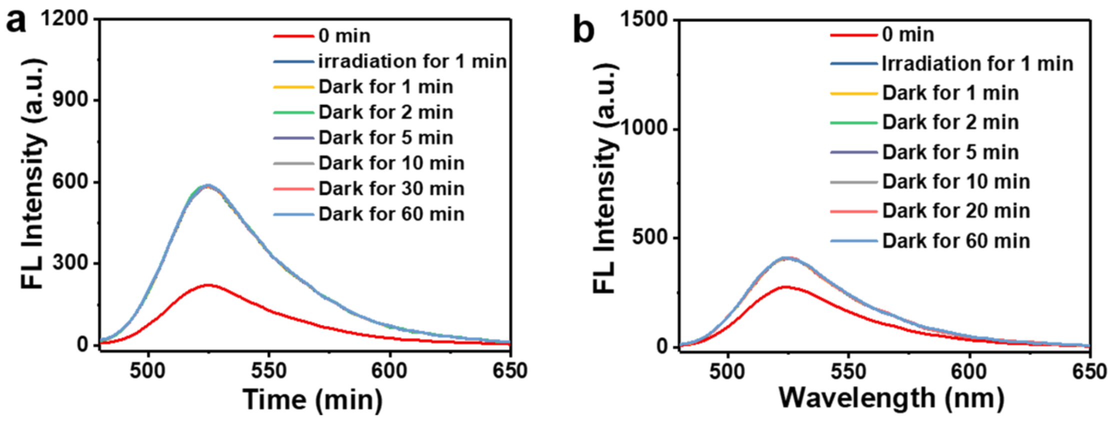

Figure S8. a) Fluorescence intensity change of SOSG in NBDP NPs (pH 5.0) triggered by 730-nm irradiation (0.05 Wcm^{-2}) for 1 min and dark for 60 min under 21% environment. b) Fluorescence intensity change of SOSG in NBDP NPs (pH 5.0) triggered by 730-nm irradiation (0.05 Wcm^{-2}) for 1 min and dark for 60 min under 2% environment.

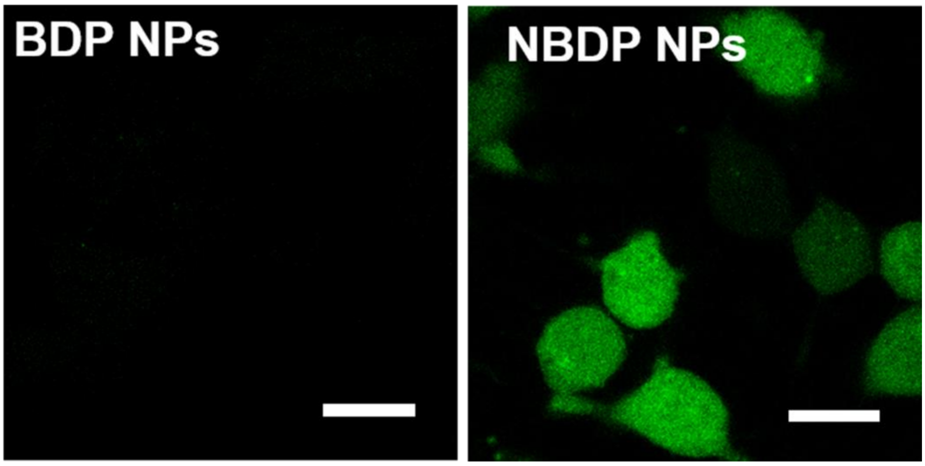

Figure S9. Intracellular $^1O_2$-photogeneration of BDP NPs, NBDP NPs. Scale bars: 10 μm.

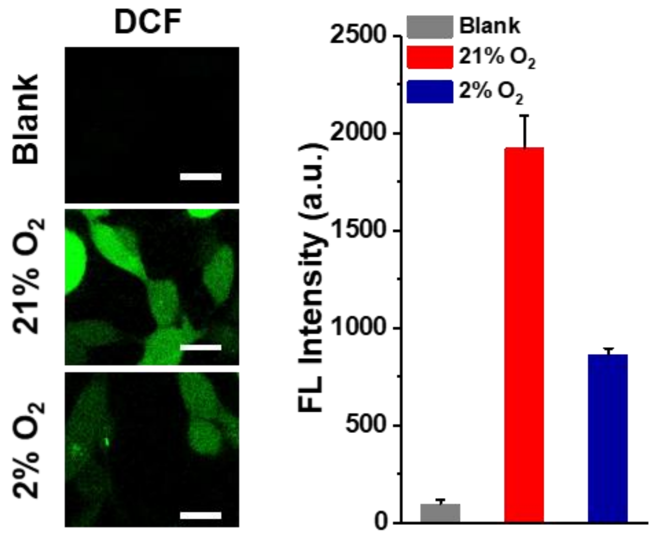

Figure S10. Intracellular $^1O_2$-generation of incubated with ANBDP NPs in the light state under normoxia (21% O_2) and hypoxia (2% O_2). Blank group indicated cells was incubated with no ANBDP NPs under normoxia. Scale bars: 10 μm.

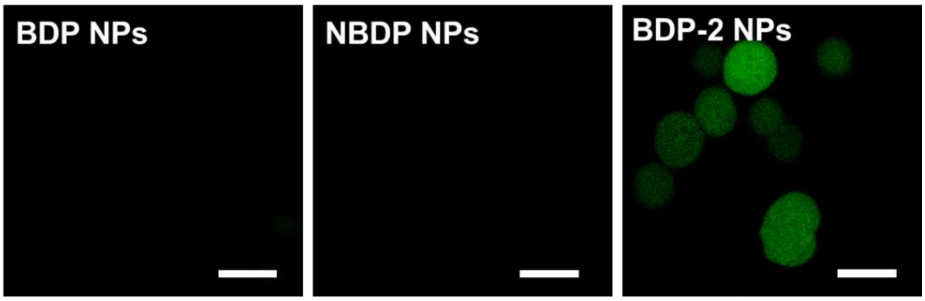

Figure S11. Intracellular $^1O_2$-generation in the dark state triggered by BDP NPs, NBDP NPs, and BDP-2 NPs under 21% O_2. Scale bars: 10 μm.

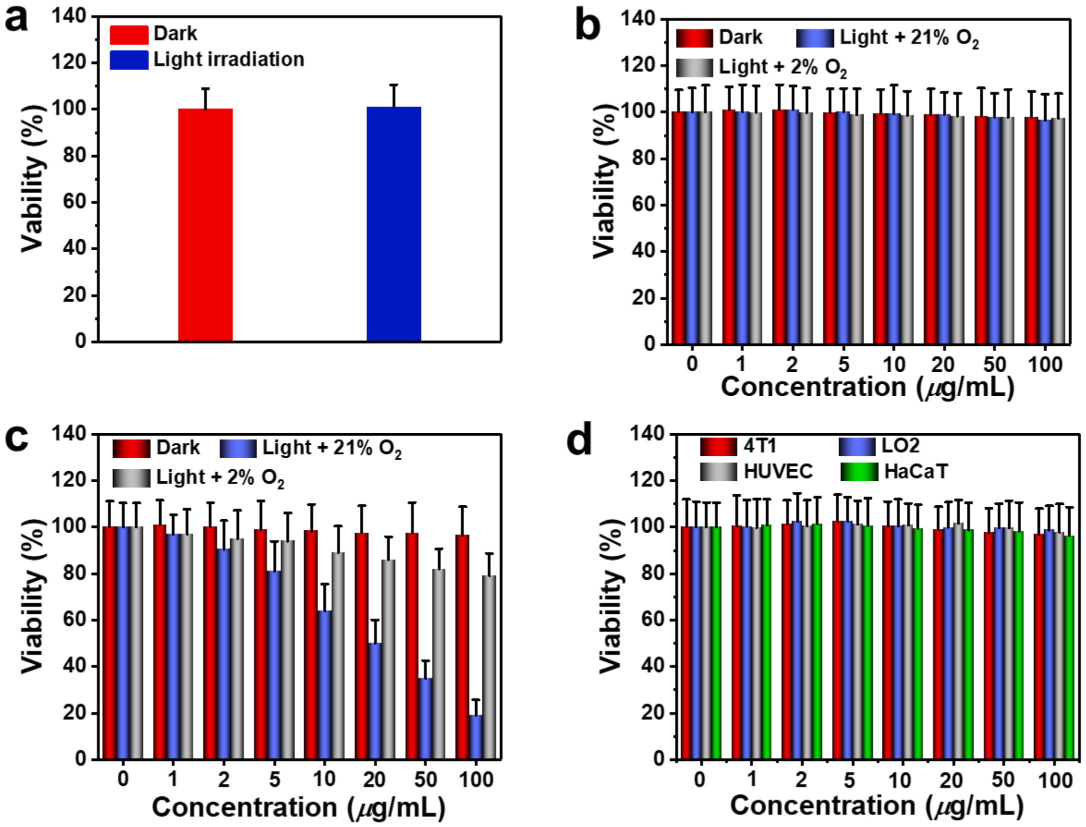

Figure S12. a) Viability of 4T1 cells with or without irradiation. b) MTT assays on 4T1 cells treated with different concentrations of BDP NPs under dark, light + 21% O_2, and light + 2% O_2. c) MTT assays on 4T1 cells treated with different concentrations of NBDP NPs under dark, light +21% O_2, and light + 2% O_2. d) Dark toxicity of ANBDP NPs to 4T1 cells, LO2 cells, HUVECs, and HaCaT cells.

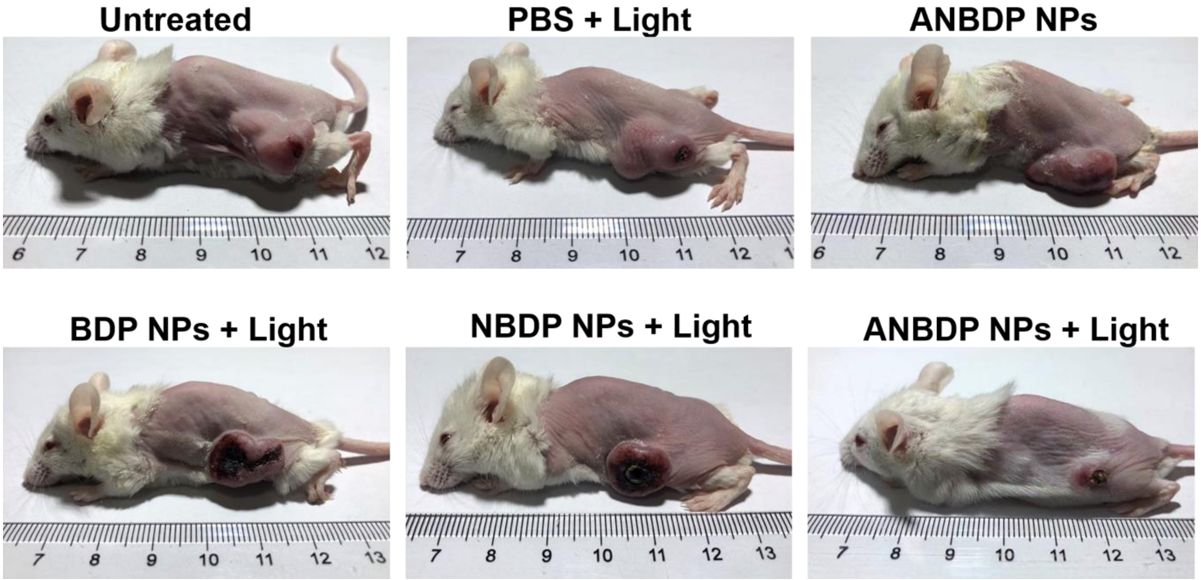

Figure S13. Photographs of mice after receiving 18 days of different treatment.

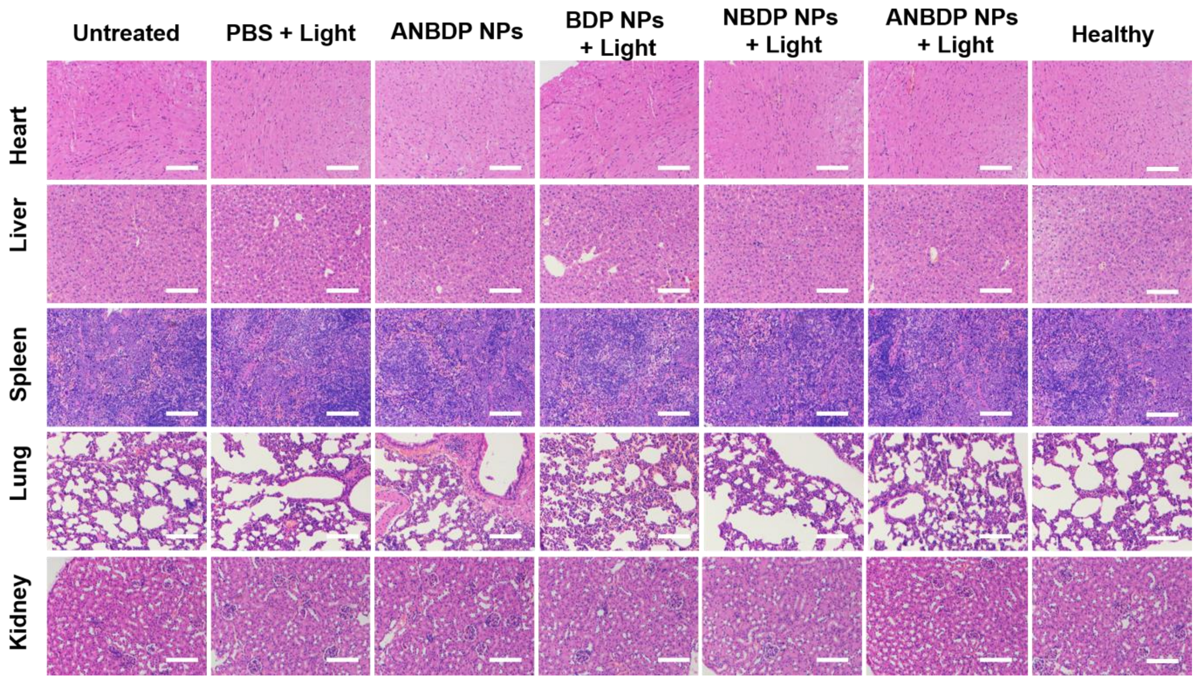

Figure S14. H&E staining results of normal organs (heart, liver, spleen, lung, and kidney) in different groups.

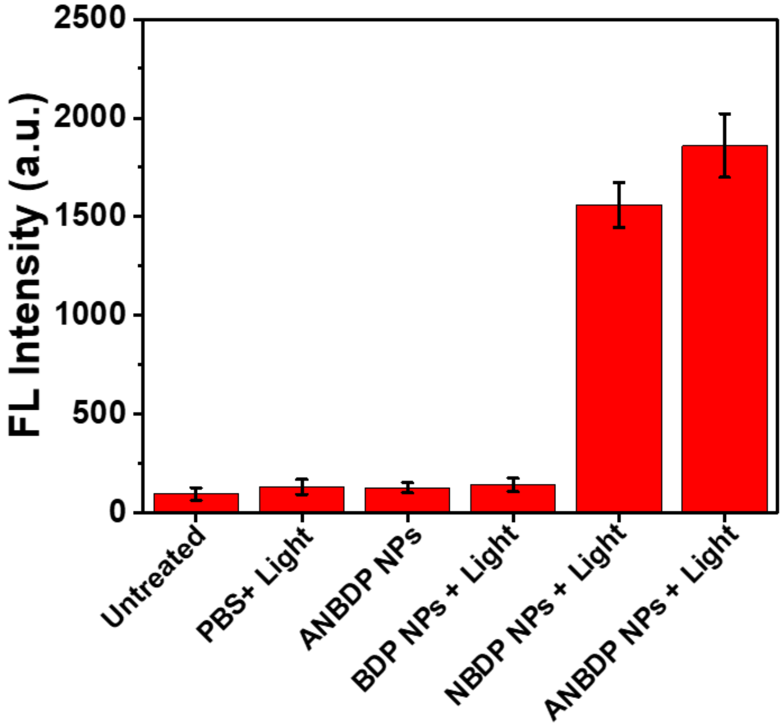

Figure S15. The quantitative FL intensity of caspase 3 in Figure 5e.

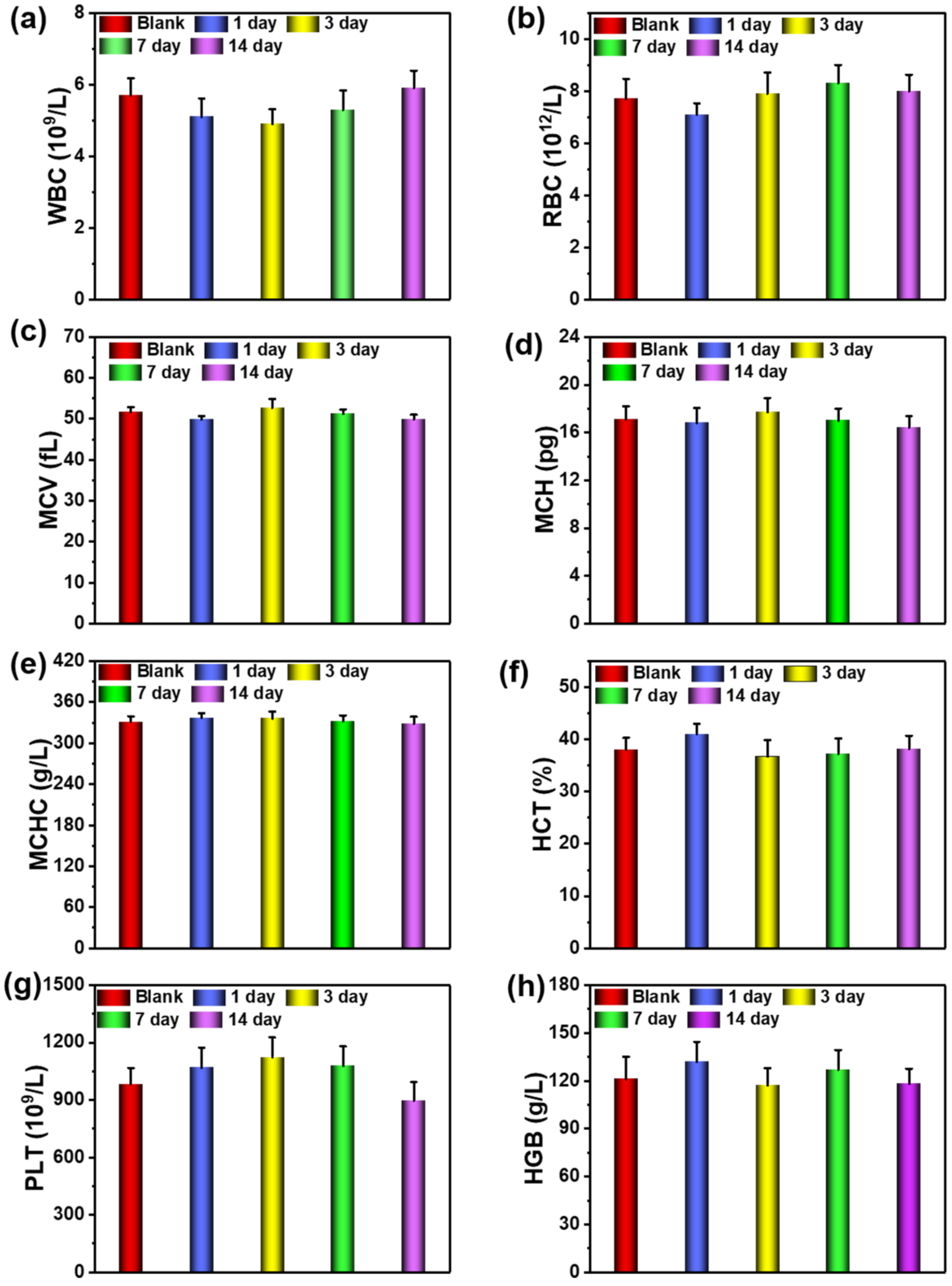

Figure S16. Blood routine examination for a) WBC, b) RBC, c) MCV, d) MCH, e) MCHC, f) HCT, g) PLT, and h) HGB in two weeks.

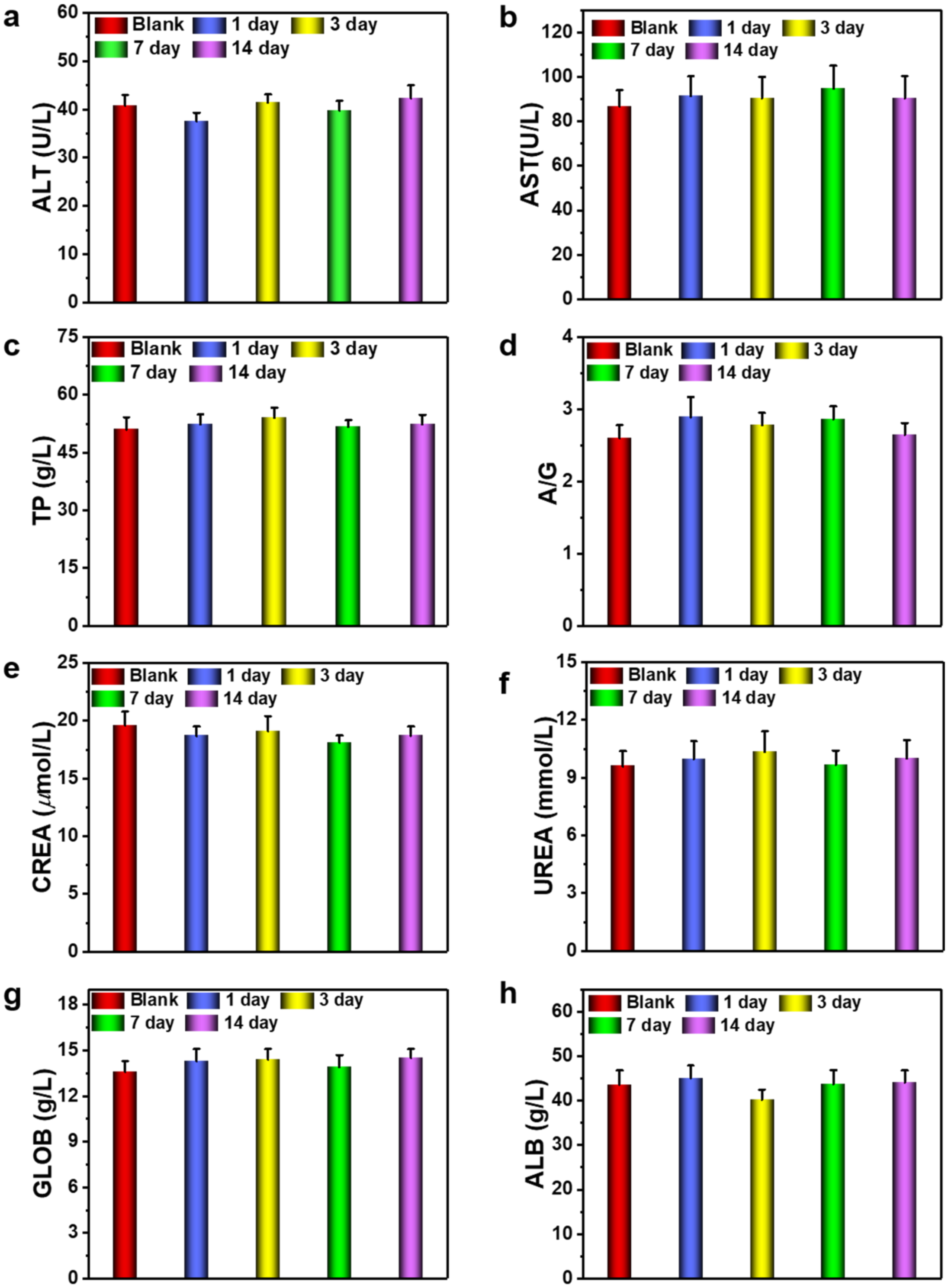

Figure S17. Serum analysis for a) ALT, b) AST, c) TP, d) A/G, e) CREA, f) UREA, g) GLOB, and h) ALB in two weeks.

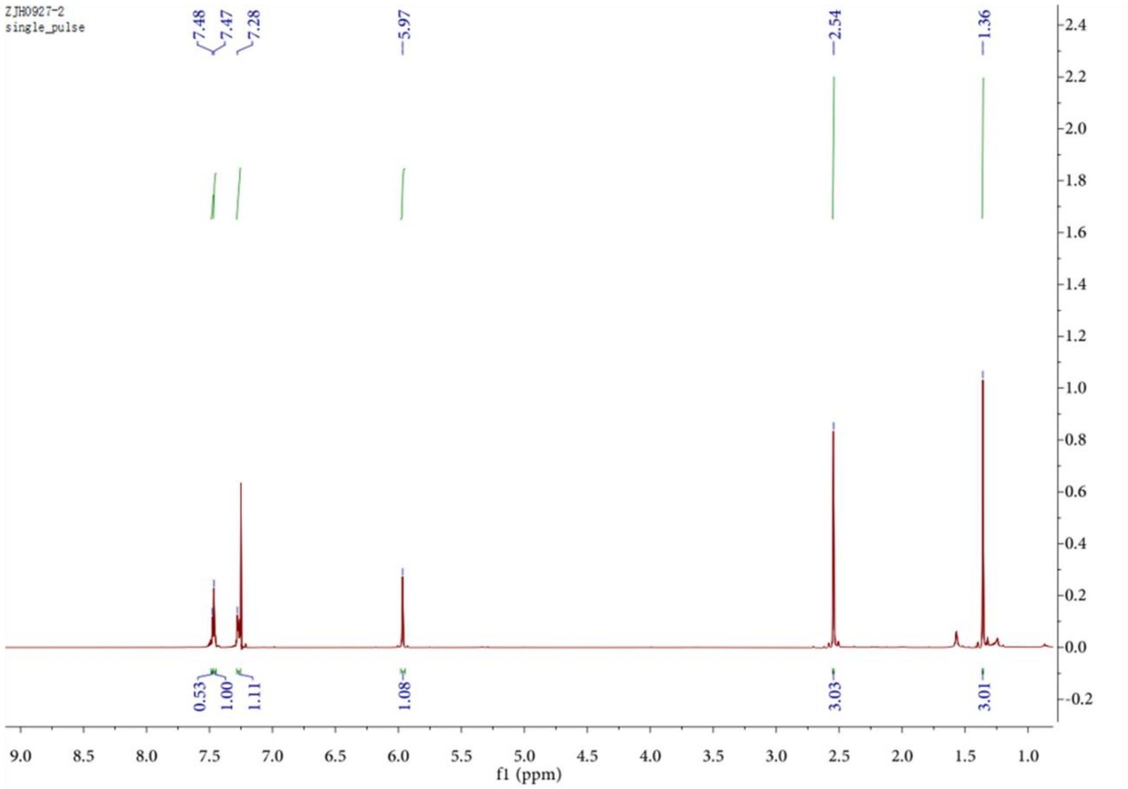

Figure S18. ^{1}H NMR spectrum of BDP-1.

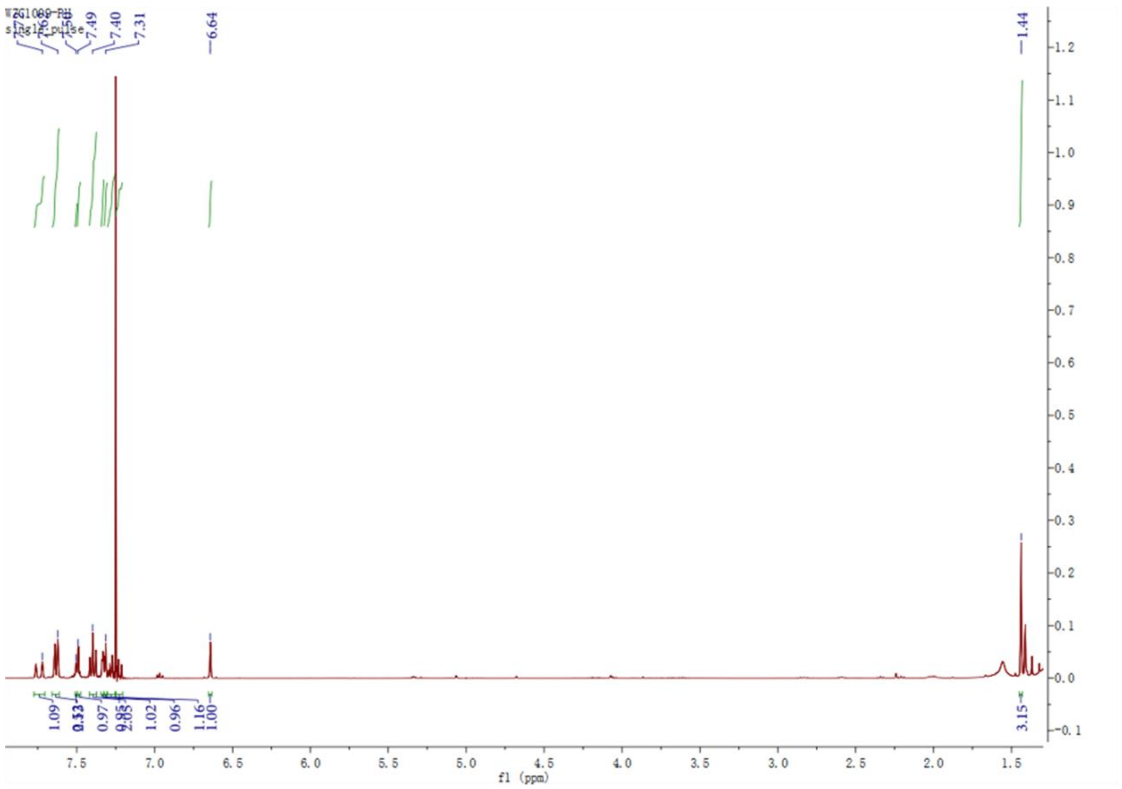

Figure S19. ^{1}H NMR spectrum of BDP.

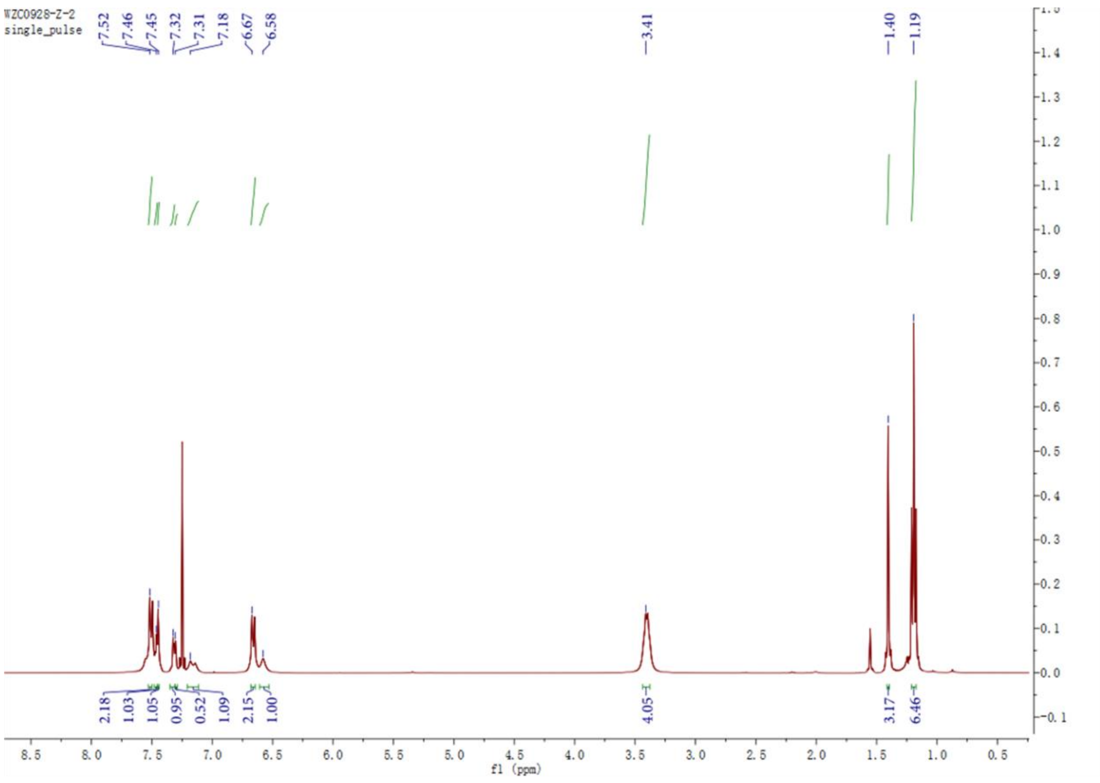

Figure S20. ^{1}H NMR spectrum of NBDP.

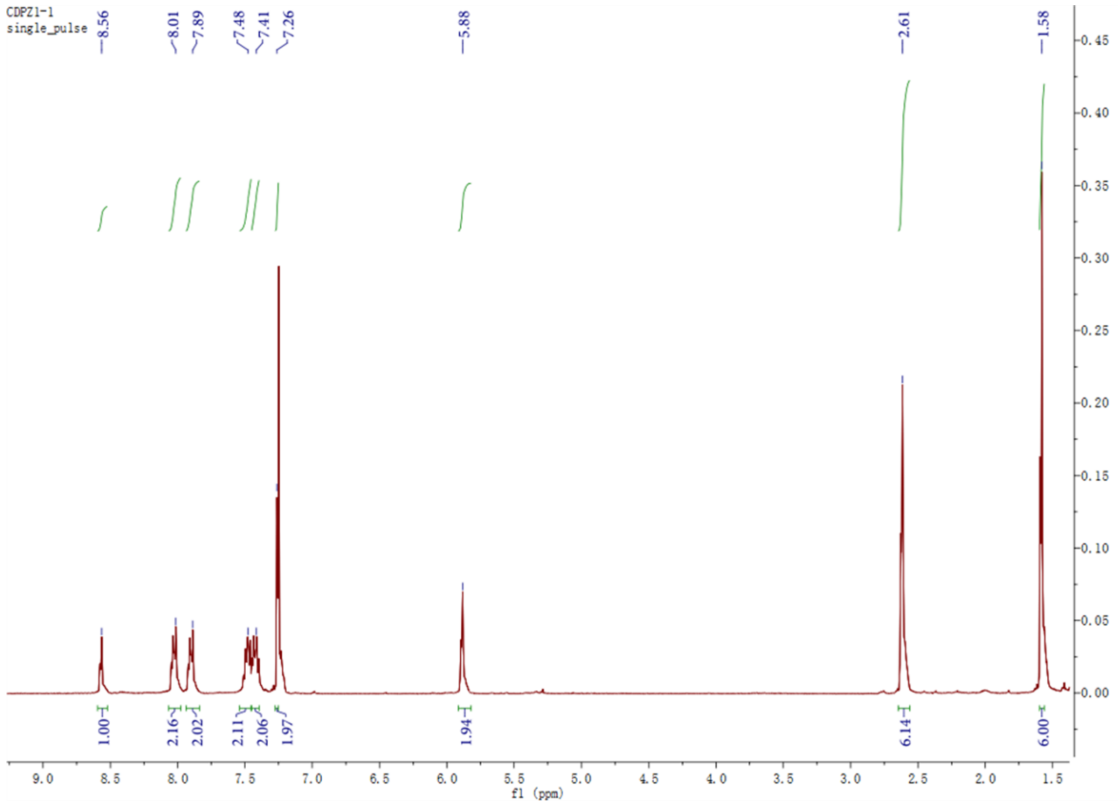

Figure S21. ^{1}H NMR spectrum of BDP-2.

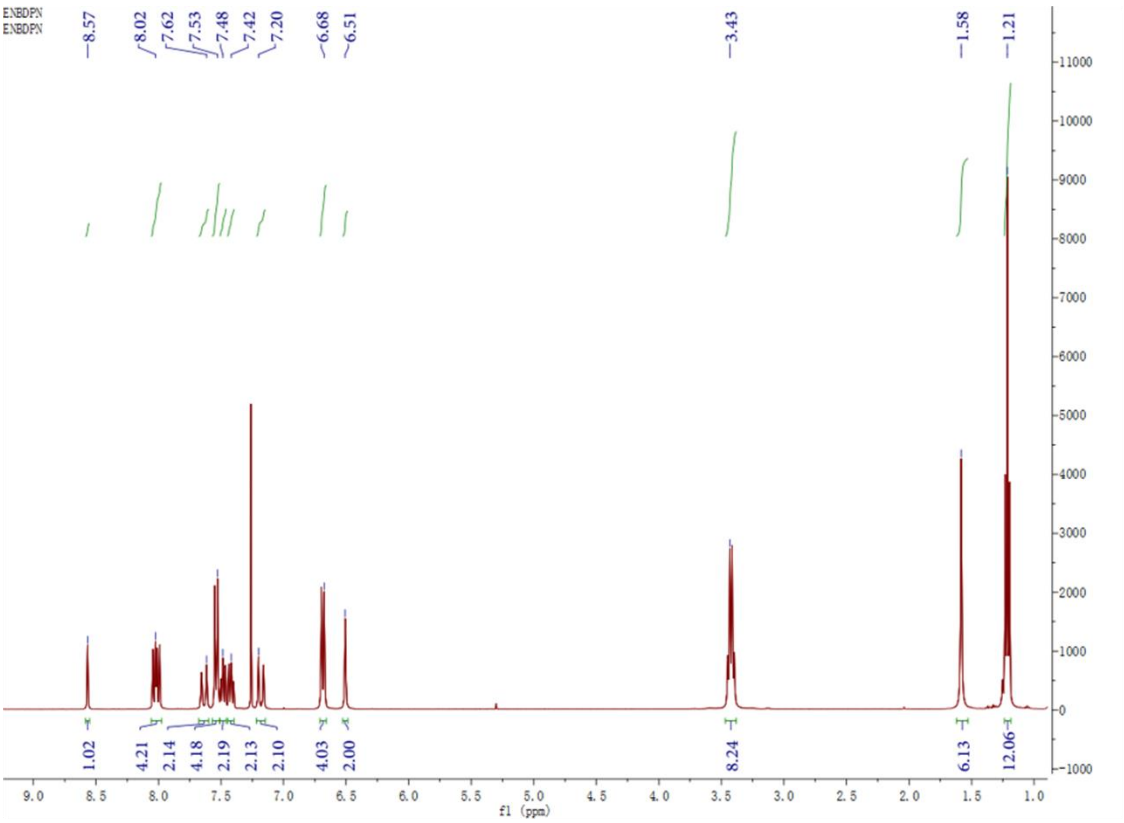

Figure S22. ^{1}H NMR spectrum of ANBDP.

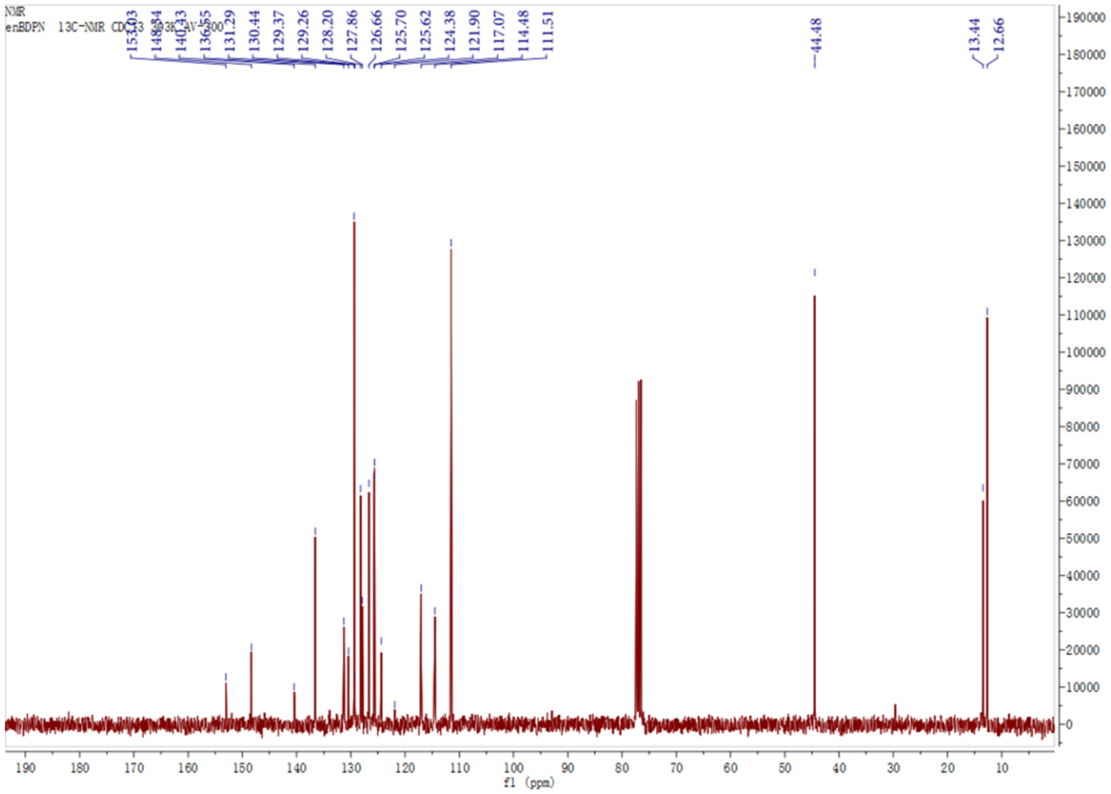

Figure S23. ^{13}C NMR spectrum of ANBDP.

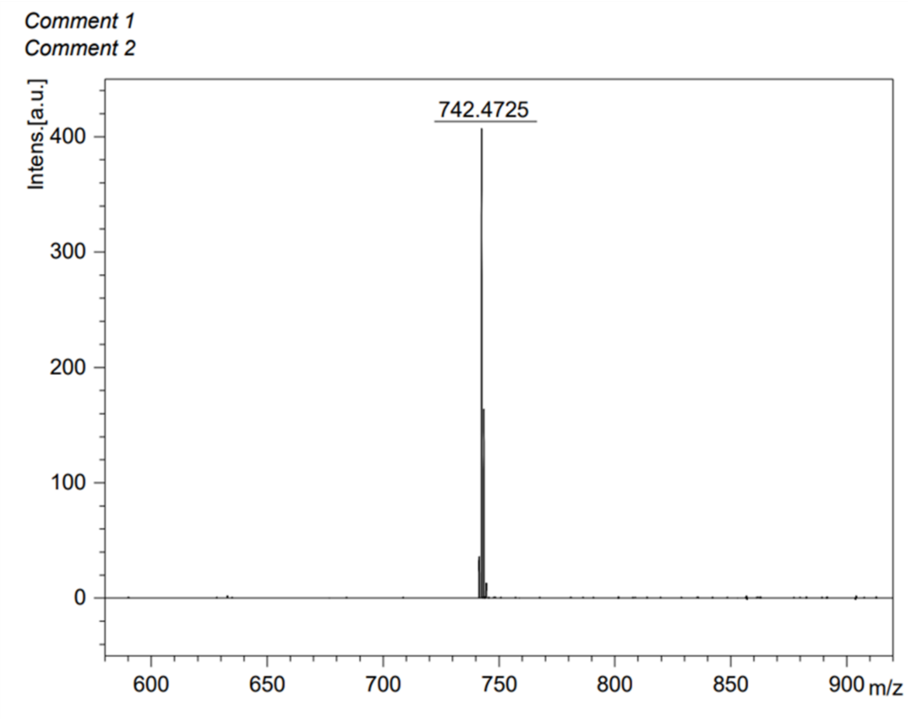

Figure S24. MALDI-TOF mass spectrum of ANBDP.
